# The German Panel of Teacher Education Students: Surveying (Prospective) Teachers from Higher Education into Working Life

**DOI:** 10.5334/jopd.76

**Published:** 2023-05-10

**Authors:** Hilde Schaeper, Andreas Ortenburger, Sebastian Franz, Stefanie Gäckle, Claudia Menge, Ilka Wolter

**Affiliations:** 1 German Centre for Higher Education Research and Science Studies, DE; 2 Leibniz Institute for Educational Trajectories, DE

**Keywords:** Teacher education, preparatory service, teachers, panel data, Germany

## Abstract

This paper describes the design, survey instruments, data, and their potential for use of a longitudinal study of (prospective) teachers in Germany that follows their professional and competence development from teacher education into the first years in the teaching profession. The Panel of Teacher Education Students (*Lehramtsstudierenden-Panel* (LAP)) is linked to the Starting Cohort 5 of the National Educational Panel Study (NEPS), which initially included about 18,000 first-year students in the winter term 2010/2011 and an oversampling of teacher education students (about 5,500 students). From 2014 onwards, multiple survey instruments—for example, aspects of preparatory service and of professional competence, instructional practices, and professional development— were specifically addressed to (prospective) teachers. The data was collected in 19 waves between 2010 and 2022.

## 1 Background

This paper describes the design, survey instruments, data, and their potential for use of the German Panel of Teacher Education Students (*Lehramtsstudierenden-Panel* (LAP)). This study is a longitudinal study of (prospective) teachers in Germany that follows their professional and competence development from the very beginning of teacher training through to preparatory service and into professional life. It is a collaborative project of the Leibniz Institute for Educational Trajectories (*Leibniz-Institut für Bildungsverläufe*; LIfBi) and the German Centre for Higher Education Research and Science Studies (*Deutsches Zentrum für Hochschul- und Wissenschaftsforschung*; DZHW). The project has been funded by the Federal Ministry of Education and Research (*Bundesministerium für Bildung und Forschung*; BMBF).

The study is closely intertwined with Starting Cohort First-Year Students (SC5) of the National Educational Panel Study (NEPS; Blossfeld & Roßbach, 2019; Brachem et al., 2019), a panel study that started in 2010 and accompanies first-year students from the winter term of 2010/2011 onwards and well into their careers.^1^ When drawing the sample, teacher education students were considerably oversampled, thus preparing the ground for the LAP study. In terms of design, the LAP study is fully integrated into NEPS SC5; in terms of constructs and survey instruments, it has complemented NEPS SC5 since 2014 (wave 8). In total, data were collected in 19 panel waves conducted between 2010 und 2022.

The main objective of the LAP is to improve the data basis for research on teacher education and the professional activities of teachers in Germany by (1) taking a longitudinal perspective (see Chapter 2.1), (2) drawing a large sample that covers the entire range of teacher education programmes in all German states (see Chapter 2.4), (3) addressing a broad range of topics (see Chapter 2.5), (4) allowing for comparisons with students and professionals in non-teaching areas, and (5) making the data available to the scientific community (see Chapter 3). The primary research interest underlying data collection is to describe and explain the development of teachers’ professional competencies and educational practices.

Central to this research focus is a theoretical approach that considers (prospective) teachers’ competencies, professional development, and teaching activities to be the result of the interplay between individual attributes of (prospective) teachers and the provision and use of learning opportunities. Following this opportunity-use model (Fend, 2006; Helmke, 2012), we measured both individual attributes of the panel members such as personality, interests, and self-concept (cf. Wohlkinger et al., 2019) and the provision and characteristics of learning opportunities. The assessment of the quality of learning environments is based on the so-called SSCO model (Bäumer et al., 2019; Schaeper & Weiß, 2016). This theoretical approach is applied throughout the NEPS and distinguishes four dimensions: structure (S), support (S), challenge (C), and orientation (O).

The concept of competence used in the LAP study follows Baumert and Kunter’s (2013) multidimensional competence model, which includes both cognitive (e.g., professional knowledge) and non-cognitive (e.g., beliefs, motivation) dimensions. Since it was not possible to measure teachers’ professional knowledge, the study focuses on beliefs (e.g., beliefs about teaching and learning), motivational orientations (e.g., motivation for choosing teacher education, teaching-related self-efficacy), and occupational self-regulation. However, as part of the NEPS SC5 testing programme, basic domain-specific competencies and domain-general or meta-competencies such as ICT literacy and social competencies (cf. Weinert et al., 2019) were assessed (see Chapter 2.5.2). This data can be, and has been, used to answer teacher-related questions, such as the level of ICT literacy (Senkbeil et al., 2021) or the impact of social competencies (Carstensen & Klusmann, 2021). In addition, data on teaching-related abilities in information and communication technologies (ICT) was collected using self-report instruments.

When assessing professional competencies, the LAP study measures both general aspects (e.g., professional self-concept for teaching, enthusiasm for teaching) and specific aspects. Given the challenges associated with each of inclusive teaching, increasingly multicultural classes, and the growing importance of ICT for teaching and learning, the study places a special emphasis on these three areas and gathers information, for example, on inclusion-related, multicultural, and ICT-related beliefs. In order to provide an opportunity to expand knowledge on the effects of these competencies and the driving factors behind them, we collected data on learning opportunities for teaching in inclusive and multicultural classes and also on several teaching practices. Using this data, Menge et al. (2021) examined the effect of learning opportunities and experiences on (prospective) teachers’ beliefs towards inclusion and self-efficacy regarding inclusive teaching.

The LAP study addresses all stages of teacher formation. First, the initial phase in higher education: Although it was not possible to include specific teacher-related questions in the survey programme before 2014, the comprehensive basic surveys of NEPS SC5 open up the opportunity to examine many research questions on teacher candidates and teacher education. Complementing previous research on specific characteristics of teacher education students, the large and diverse sample of the LAP study allows for a more differentiated view, which has already been taken, for example, by Hartmann and Ertl (2021), Hartmann et al. (2022), Neugebauer (2020), and Osada and Schaeper (2021), or to focus on specific groups, such as students with a migrant history (cf. Besa & Vietgen, 2017; Gülen, 2021, 2022). Other questions, which can be analysed using the core data of NEPS SC5, refer to the learning experiences (see the paper of Rochnia et al, 2019) and to the factors that influence educational choices during higher education.

The second stage of teacher training, the preparatory service or induction phase, combines practical training in schools with studies in educational theory and subject-related didactics at seminars, and concludes with a state examination. Since this *Referendariat* or *Vorbereitungsdienst* is considered a crucial phase of teacher education, which is often associated with high levels of occupational stress (Drüge et al., 2014; Klusmann et al., 2012), the LAP study gathers information on relevant aspects of this learning environment.

‘Learning while working as a teacher’ constitutes the third stage of teacher education (Fussangel et al., 2016). This stage includes professional development (PD) activities and continuing education in formal and nonformal learning environments but also learning in informal settings (for the distinction between different types of learning contexts see Bäumer et al. (2019)). Following the model of Richter et al. (2010) for explaining participation in continuing education, the LAP study collects information on context-specific, social, psychological, and situational factors.

Not only do colleagues and school leaders play a role in individual decisions to take advantage of continuing learning opportunities, but colleagues also offer informal learning opportunities, and colleagues and the school management are elements of the school context that influences teaching and teachers’ learning (Lipowsky & Rzejak, 2019). The LAP study measures cooperation among colleagues as perceived by the individual teacher using a three-stage model that distinguishes between exchange, joint work, and co-construction (Dizinger & Böhm-Kasper, 2019; Gräsel et al., 2006).

Through their leadership style school leaders can have a significant impact on the school climate and culture, and in this way also on the individual teachers’ motivation, beliefs, well-being, and instructional practices (Blömeke & Klein, 2013; Leithwood & Sun, 2012; Pietsch & Tulowitzki, 2017; Windlinger et al., 2020). The LAP study collects information on two widely adopted (Pietsch & Tulowitzki, 2017) and highly predictive (see the meta-analyses of Hattie, 2009; Judge & Piccolo, 2004; Robinson et al., 2008; Sturm et al., 2011) concepts: instructional leadership, which is learning- and teaching centred (Bush & Glover, 2014; Pietsch & Tulowitzki, 2017), and transformational leadership, which is directed towards “building up the capacity of those they work with, motivating them towards instilling change and transformation” (Pietsch & Tulowitzki, 2017, 632).

Finally, the LAP study addresses “alternatively certified” teachers, who have come into the focus of policy and research because of teacher shortage, especially in STEM subjects. Therefore, Germany has opened up alternatives to the traditional route into the teaching profession. Under certain conditions, it is possible to work as a teacher without a teaching-related higher education degree; either with a completed preparatory service (*Quereinstieg*) or without having undertaken preparatory service (*Seiteneinstieg*; cf. Lucksnat et al., 2020).

The variety of topics briefly mentioned above shows that the LAP data can be used to address a wide range of research questions, particularly in educational psychology. More details can be found in Chapter 4.

## 2 Methods

### 2.1 Study design

As mentioned above, the main general objective of the NEPS is to collect longitudinal data on the development of competencies, educational processes, educational decisions, and returns to education in formal, nonformal, and informal contexts throughout the life span. In order to make relevant information on educational transitions available as soon as possible, the educational biography has been divided into eight stages with a particular focus on critical transitions. Within these eight stages, the NEPS started with six different cohorts in order to observe educational careers and transitions. This multicohort-sequence design (Blossfeld et al., 2009; von Maurice et al., 2016) makes it possible to collect comparative data on competence development and educational pathways over the entire life course, while at the same providing the data in a relatively short timeframe. Theoretical dimensions, so-called ‘pillars’, provide a conceptual framework and integrate data collection in these six starting cohorts.

One of the starting cohorts mentioned refers to the stage of higher education. For this cohort, first-year students, enrolled at a German higher education institution (HEI) in the winter term of 2010/2011, were selected (Aßmann et al., 2019). HEIs include universities and equivalent institutions such as colleges of art or music, as well as universities of applied sciences. Students attending private HEIs and students enrolled in a teaching degree programme were oversampled. As mentioned above, the latter form the basis of the LAP project. For details on the sampling design see Chapter 2.4.

In Starting Cohort First-Year Students several modes of data collection were employed: self-administered paper-and-pencil questionnaires (PAPI), computer-assisted telephone and personal interviewing (CATI and CAPI), online surveys (computer-assisted web interviewing; CAWI), and competence testing.

Following the initial PAPI in the winter term of 2010/2011, annual CATIs (and one CAPI in wave 12) focus primarily on (an update of) the life course, including the educational and employment history of the participants. Central longitudinal measures of the NEPS pillars can be found in CATI/CAPI as well as in CAWI, which are conducted annually from 2011 until 2014 and biennially thereafter. In addition, questions particularly aimed at higher education students are integrated into the CAWIs. All survey instruments were basically also addressed to (prospective) teachers. They already open up a wide range of possibilities for research on teacher training and the teaching profession. When the LAP project started, an additional survey programme specifically for (prospective) teachers has been implemented in the NEPS SC5 surveys since wave 8 in autumn 2014, with the LAP study fully integrated into the survey design.

The data collected alongside the life course information within a wave can be interpreted as cross-sectional data. However, since the central constructs are usually collected in more than one wave, this data often contains longitudinal information that makes it possible to observe intra-individual developments.

A variety of modes were used for the competence test (Brachem et al., 2019). The first competence test in wave 1 was administered as a paper-based assessment (PBA). For the second test of competencies in wave 5, a mode experiment was conducted with individual web-based testing (CBWA), as well as three test modes in group settings: conventional paper-based assessment (PBA), paper-based assessment with digital pencils (E-Pen), and computer-based assessment (CBA). In wave 7, a subject-specific competence test in business administration was administered as an individual paper-and-pencil test to students or graduates of business administration and economics. The last competence test of NEPS SC5 was conducted in wave 12, again as a mode experiment in two modes: CBA during a CAPI survey and CBWA after a CATI survey (for more details, see the ‘Information on Competence Tests’ and ‘Field Reports’ at: https://www.neps-data.de/Data-Center/Data-and-Documentation/Start-Cohort-Students/Documentation).

### 2.2 Time of data collection

Between 2010 and 2022, 19 panel waves were conducted. The CATIs with their focus on the life course always started in spring, with the online surveys usually starting in autumn. Only the nineteenth wave, in 2022, deviates somewhat from this pattern; wave 19 is a combined CATI and CAWI survey, where all study participants who took part in the telephone interview were invited to the online survey immediately afterwards. Although unscheduled, the study participants of all NEPS starting cohorts were also asked online about the effects of the Corona pandemic in May/June 2020.^2^ A detailed list of the studies conducted can be found in Table 1. The table also shows when the competence tests took place. The data published to date comprises data from the first 17 panel waves and the additional 2020 Corona survey.

**Table 1:** Overview of surveys and tests.


WAVE	MODE/METHOD	START	END	COMMENT

1	PAPI (recruitment survey) & CATI	30.11.2010	28.01.2012	

1T	Competence test	21.03.2011	22.07.2011	Group administered PBA

2	CAWI	26.10.2011	11.12.2011	

3	CATI	10.04.2012	18.08.2012	

4	CAWI	29.10.2012	17.12.2012	

5	CATI	19.03.2013	03.08.2013	

5T	Competence test	02.05.2013	31.07.2013	Mode experiment: group administered PBA/E-Pen/CBA & individual CBWA

6	CAWI	29.10.2013	15.12.2013	

7T	Competence test	24.01.2014	06.04.2014	Individual subject-specific PBA

7^a)^	CATI	28.04.2014	13.09.2014	

8^b)^	CAWI	29.10.2014	07.12.2014	

9	CATI	27.04.2015	31.08.2015	

10	CATI	21.03.2016	07.08.2016	

11	CAWI	02.11.2016	11.12.2016	

12	CAPI CATI	27.02.2017 25.04.2017	04.08.2017 23.09.2017	Mode experiment: CAPI/CATI

12T	Competence test	27.02.2017	30.11.2017	Mode experiment: individual CBA and CBWA

13	CATI	23.04.2018	01.09.2018	

14	CAWI	07.11.2018	16.12.2018	

15	CATI	18.03.2019	03.08.2019	

16	CATI	16.03.2020	01.08.2020	
	CAWI	13.05.2020	22.06.2020	Additional NEPS survey on the Corona pandemic, published since version 14.1.0 of the Scientific Use File (SUF)

17	CAWI	04.11.2020	11.01.2021	

18^c)^	CATI	22.03.2021	21.08.2021	

19	CATI + CAWI	19.04.2022	17.09.2022 13.11.2022	Combined CATI and CAWI survey

a) Without the LAP oversample; b) Additional questions specific to teaching for the first time (see Chapter 2.1 and 2.5); c) For internal reasons, the field period ended for the LAP oversample on 19.06.2021 and the additional questions specific to teaching were suspended.				

Please note that for funding reasons the initial sample of teacher education students was randomly divided into a “LAP basic sample” and a “LAP oversample”. The relative size of the basic sample corresponds to the proportion of teacher education students in the population, and the size of the oversample is the surplus. The two groups were sometimes treated differently.

### 2.3 Location of data collection

All data was collected across all German federal states. In addition, study participants who belong to the initial sample were also surveyed abroad when they moved to another country.

### 2.4 Sampling, sample and data collection

The target population of NEPS Starting Cohort First-Year Students consists of all students who enrolled at a public or state-approved HEI in Germany for the first time in the winter term of 2010/2011 and were aiming to complete one of the following degrees: bachelor’s degree, a state examination (*Staatsexamen*) in medicine, law, pharmacy, or teaching, or a diploma or master’s degree in theology. However, first-year students studying at higher education institutions run by federal ministries or federal states for members of their own public services were excluded. During sampling, special emphasis was placed on students with non-traditional entrance qualifications, i.e., students without a school-leaving certificate qualifying for higher education. Furthermore, students at private HEIs and teacher education students were overrepresented in the sample, the latter building the initial sample of the LAP. More details on the population and sampling are described in Zinn et al. (2017) and Aßmann et al. (2019).

The sample for the first wave was drawn using a stratified cluster sampling approach. A particular field of study at a specific higher education institution represented one cluster (primary sampling unit), and all students within each cluster were surveyed. To oversample teacher education students (and students at private HEIs), a first stratification level distinguished between clusters in terms of type of HEI, institutional control of HEI, and teacher education programme. This first stratification level consisted of four strata (*h*_1_ = clusters linked to teaching tracks; *h*_2_ = all other fields of study at public universities; *h*_3_ = all other fields of study offered at public universities of applied sciences; *h*_4_ = all study tracks at private universities or private universities of applied sciences). To reduce sampling error, a second stratification level was introduced, which was defined using aggregated fields of studies. A detailed report of the stratified cluster sampling approach as well as a detailed description of the clusters and strata can be found in Zinn et al. (2017).

In order to achieve higher response rates, two separate approaches were used for recruiting study participants: In a first step, each student was invited by letter to participate. In a second step, field workers were sent into central first-year courses to personally recruit participants for the survey. This method had been previously tested in a pilot study and resulted in both better participation rates and higher panel attendance (Brachem et al., 2019).

In the first wave, a total of 17,909 first-year students participated in the NEPS student cohort including 5,554 teacher education students (according to variable tg02001; see Chapter 3.10) who form the initial LAP target group. The various school types of the German school system are also mirrored in different teacher education programmes. In the initial sample, 13% of the teacher education students had taken up a training course for primary education, 19.5% for lower secondary education (excluding lower secondary education at a *Gymnasium*), 54.5% for upper secondary education (including lower secondary education at a *Gymnasium* but excluding vocational education), 5% for special education, and 6.5% for vocational education. One and a half per cent of the teacher education students stated that their degree programme did not differentiate between school types. As is common in teacher education programmes, most students are female (75.5%).

However, educational and occupational trajectories are not linear, and dropping out or changing study programmes is common. Hence, the target population changes over time due to people dropping out of or moving into teacher education, or entering the teaching profession without having completed a teaching degree (e.g., lateral entrants). The LAP target population was initially limited to teacher education students, trainee teachers in preparatory service and working teachers. From wave 11 onwards, the definition of the target population was expanded to include respondents who (a) have completed preparatory service and intend to work as teachers, but are not yet employed, (b) have completed the first phase of teacher education and intend to complete preparatory service but have not yet started it, and (c) have temporarily interrupted employment as a teacher, e.g., due to parental leave.

Table 2 reports the number of participants from wave 8 to wave 17 who belong to the LAP target group. The table also shows the auxiliary variables generated from wave 8 onwards to filter the teacher-specific questions and determine the LAP sample. More information on identifying the LAP target population, also in earlier waves, and auxiliary variables provided for this purpose is given in Chapter 3.10. A full description of the student cohort’s recent panel development can be found in Ziesmer (2022).

**Table 2:** Size of the LAP target group by status (wave 8 to wave 17).


WAVE	VARIABLE	TEACHER EDUCATION PROGRAMME	PREPARATORY SERVICE	EMPLOYED AS TEACHER	TOTAL

8^a)^	tg60011	2,757			2,757

9^b)^	tg60014 & tg64012	2,870	341		3,211

10	tg60022_v1, tg60015 & tg64012	1,679	857	282	2,818

11	tg60012_v1	905	776	291	1,972

12	tg60013_v1	823	1,042	691	2,556

13	tg60013_g1v2	420	686	1,023	2,129

14	tg60017_v1	222	304	840	1,366

15	tg60013_g1v2	244	318	1,279	1,841

16	tg60013	135	152	1,444	1,731

17	tg60017	109	82	1,197	1,388

a) Information on participants in preparatory service or working as teachers not shown separately; b) Information on participants working as teachers not shown separately.					

The analysis shows that participation rates are consistently lower in CAWI compared to CATI. The proportion of students who could be reached for an interview or test declined significantly over time. The main reasons for panel attrition are missing contact information, continuous non-participation over a period of three years (so-called ‘final dropouts’), and withdrawal of panel consent (Aßmann et al., 2019). There is evidence that accessibility and participation of study participants depend on both the topic of the survey and personal availability as well as on socio-demographic factors (Liebeskind & Vietgen, 2017; Zinn et al., 2018). To account for selective study participation, weights have been estimated for every wave. More details on participation development, panel attrition, sample selectivity and weights can be found in Zinn et al. (2018) and, for the most recent Scientific Use File (SUF), Ziesmer (2022).

### 2.5 Materials and survey instruments

#### 2.5.1 Materials

The survey instruments of all published waves as well as other information materials are well documented and publicly available. The following list describes some of the main sources of information and documentation materials for data users (see also Table 4):

**Table 3:** Overview of NEPS SC5/LAP datasets (as of February 2023).


DATA FILE^a)^	CONTENT

Basics	Basic information about participants, such as sociodemographic characteristics

Biography	Integrated and edited life course data; serves to facilitate the analysis of complex life course data

CohortProfile	Meta information for all participants and waves, such as participation status and interview date

EditionBackups	Information on single values that have been changed or modified in the data edition process

Education	Longitudinal information on transitions in participants’ educational history

MethodsCATI	Paradata from the CATI interviews, e.g., interview duration, interviewer characteristics

MethodsCAWI	Paradata from the CAWI interviews, e.g., interview duration

MethodsCompetencies	Paradata from the competence tests, e.g., test date, interviewer characteristics

pTargetCATI	Data from CATI questionnaires

pTargetCAWI	Data from CAWI questionnaires

pTargetMicrom	Small-scale regional indicators of participants’ place of residence; only available via on-site access

spChild	Information on participants biological, foster, and adopted children

spChildCohab	Data on cohabitation spells with children

spCourses	Information on courses/trainings taken during employment, unemployment, parental leave, and other episodes

spEmp	Information on employment episodes

spFurtherEdu1	Information on further courses since the last interview that have not been reported elsewhere

spFurtherEdu2	Additional information on two randomly selected course from the ‘spCourses’ and ‘spFurtherEdu1’ modules

spGap	Information on gaps in the individual life course identified by a check module (e.g., type of gap)

spInternship	Information on internship episodes that took place after completing school and before or during higher education

spMilitary	Data on episodes of military/civilian service and voluntary gap years

spParLeave	Data on parental leave episodes

spPartner	Data on the participants’ partnership history and basic information about the participants’ partners

spSchool	Data on participants’ general school history

spSchoolExtExam	Information on school certificates acquired by recognition or external examination

spSibling	Basic information on participants’ siblings reported in wave 1

spUnemp	Data on unemployment episodes

spVocBreaks	Information on breaks further education and training with a special focus on higher education

spVocExtExam	Information on vocational education certificates acquired outside the regular German educational system, e.g., certificates acquired abroad or as an external examinee

spVocPrep	Data on episodes of vocational preparation after general education

spVocTrain	Information on participants’ vocational education history covering all further (vocational and/or academic) trainings

StudyStates	Information on participants’ higher education phase or status, derived from ‘spVocTrain’; e.g., type of degree acquired, ongoing/completed degree programme

Weights	Sample weights, cluster and stratification variables

xEcoCAPI	Data of a subject-specific competence test in business administration, conducted on a subsample of students in economics

xInstitution	Context data for all higher education institutions (e.g., size, regional unemployment rate) and subject areas within institutions (e.g., number of female and male first-year students, number of professors); most variables only available on-site

xPlausibleValues	Plausible values of the competence data stored in ‘xTargetCompetencies’

xTargetCompetencies	Data from competence tests in reading (German), mathematics, science, ICT, English, and domain-general cognitive functioning

xTargetCORONA	Data collected in an additional survey in May/June 2020 regarding the impact of the COVID-19 pandemic on participants’ life

NEPS-SC5-ADIAB	Combined NEPS SC5 data and administrative data from the Institute for Employment Research (IAB); available in restricted access environments

a) The complete names of the datasets consist of (1) an indicator for the starting cohort (e.g., SC5), (2) the unique file name indicating content and type of data (e.g., Basics), (3) an indicator for the confidentiality level (D = download; R = remote access; O = on-site access) and (4) an indicator for the release version. For example, the ‘Basics’ file of NEPS SC5, download version, SUF release 17.0.0 is labelled ‘SC5_Basics_D_17-0-0’. Unique file names with the prefix ‘x’ refer to cross-sectional data, the prefix ‘sp’ indicate spell data, and the prefix ‘p’ panel data. Datasets without a prefix are generated by the LIfBi Research Data Center.	

**Table 4:** Overview of relevant documents and materials (as of February 2023).


DOCUMENT/MATERIAL	DESCRIPTION

NEPSPlorer^a)^	Tool for searching through survey instruments of all NEPS Starting Cohorts

Glossary^b)^	Overview of the NEPS terminology and abbreviations and specific terms in the German education and occupation system

Questionnaires^b)^	Provided as SUF and field versions (SUF versions in German and English)

Field reports^b)^	Information on the data collection process

Interviewer manuals^b)^	Information for the interviewers on the process and content of the interviews

Check module for life course data^b)^	Description of the module that checks the life course data reported in CATI for gaps between and overlaps of episodes

Information on competence tests^b)^	Description of the measured constructs, data, and psychometric properties

Data manual^b)^	Overview of the panel waves and data

Codebook^b)^	Overview of all variables measured

Semantic data structure file^b)^	Contains meta-data stored in the SUF; useful to explore the data

Release notes^b)^	Information on known bugs, solutions, and changes compared to previous versions

Anonymisation manual^b)c)^	Description of the anonymisation procedure; overview of restricted variables

Sample, weights, nonresponse^b)^	Information on the sampling process and the construction of the weighting variables

Merging matrix^b)^	Instruction of how to link information from different datasets

Non-traditional students^b)^	Documentation of the variable tg24150 (non-traditional students)

Context data^b)^	Description of the context data for higher education institutions (file ‘xInstitution’)

Regional data^b)^	Documentation of the regional structural information; can be merged on-site

Immigrants in the NEPS^d)^	Information on how immigrants were identified and categorised in the NEPS

Special Stata commands^e)^	NEPS-specific Stata commands in the package ‘NEPStools’

a) https://www.neps-data.de/Data-Center/Overview-and-Assistance/NEPSplorer. b) https://www.neps-data.de/Data-Center/Data-and-Documentation/Start-Cohort-Students/Documentation. c) https://www.neps-data.de/Data-Center/Data-Access/Sensitive-Information. d) Olczyk et al., 2016. e) https://www.neps-data.de/Data-Center/Overview-and-Assistance/Stata-Tools.

- The *NEPSplorer* (https://www.neps-data.de/Data-Center/Overview-and-Assistance/NEPSplorer; description in Fuß & Wenzig, 2019; Skopek et al., 2016) is a tool that “performs a full text search through the German and English survey instruments of all released Scientific Use Files with the exception of competence tests” (Fuß & Wenzig, 2019, 370).
- The *Codebook* of the most recent SUF gives an overview of all variables measured and provides information on the number of cases and measurement time points. The codebook for NEPS SC5 including the LAP data is available in German and English.
- *Questionnaires* are provided as SUF and field versions. While the field versions consist of the original paper & pencil questionnaires or programming templates, the SUF versions include additional information such as the variable names. The SUF versions of the survey instruments have also been translated into English. Survey instruments of panel waves not yet published can be accessed once having registered as a NEPS data user. However, it is not possible to get exact information on the competence tests (e.g., wording of the test items, instruction).
- The *Data Manual* is an important and useful source of information on general features of the panel waves, conventions, and data structure. In addition, it contains a chapter dealing with special issues such as special types of variables and coding strategies. In this chapter, data users will also find a special LAP subchapter. We strongly recommend that users check the data manual regularly for updates and changes made in the published SUF.
- *Field reports* give information on the process of data collection, and document—for example—tracking of panel members, incentives given to respondents, reminders, and measures taken for contacting participants. They are written by the social research institute “infas Institute for Applied Social Sciences” (*infas Institut für angewandte Sozialwissenschaft*, Bonn), which conducted most of the surveys and tests. Field reports are only available in German.

Apart from the NEPSplorer, all documentation materials described above can be downloaded from https://www.neps-data.de/Data-Center/Data-and-Documentation/Start-Cohort-Students/Documentation. Links to the documentation of previous SUFs can also be found on this page.

#### 2.5.2 Survey instruments

In the following sections, we describe the survey instruments specifically targeted to (prospective) teachers and selected survey instruments of the basic NEPS SC5 programme addressed to all panel members (see Section “Selected NEPS constructs” below). Because of the many panel waves conducted in NEPS SC5, it is not possible to give an exhaustive account of the constructs measured. However, more information on the instruments used in NEPS SC5 including theoretical frameworks is given in various chapters of the books edited by Blossfeld et al. (2016) and Blossfeld and Roßbach (2019). For clarity, we present basic information such as construct name, subscales, and sources in tabular form where appropriate, and organise the description in subsections. Information on psychometric properties is not provided but will be published shortly in the documentation of the LAP survey instruments.

Although the data of the most recent panel waves has not yet been released, we will describe the measured constructs of all waves. As described in Chapter 3.8, this data will be published in the foreseeable future.

##### Selected NEPS constructs: Aspects related to studying and the course of studies, personality, motivation, and well-being

As in other NEPS starting cohorts, data collection in NEPS SC5 revolves around the question of competence acquisition and development in formal and nonformal/informal learning environments, educational decisions and transitions, their determinants and consequences, and monetary as well as nonmonetary returns to education (Brachem et al., 2019). To this end, the study assesses domain-general cognitive abilities (figural reasoning, perceptual speed), basic domain-specific cognitive competencies (German-language competencies, mathematical literacy, scientific literacy, English-language competencies), meta-competencies (ICT literacy) and social competencies using competence tests (Weinert et al., 2019). In NEPS SC5, in addition, a subject-specific competence test in business administration has been developed and was administered in wave 7 (Brachem et al., 2019; Lauterbach, 2016).

A central feature of the NEPS is the life course approach. The NEPS, therefore, records and updates the individual biography in different life spheres (e.g., schooling, vocational training (including higher education), employment, family and partnership) and provides detailed event-history data for these domains. In this way, the complete course of study is recorded in great detail, including moves to another higher education institution, change of subject, and change of degree. In addition, the NEPS collects information on learning environments, academic performance, psychological factors, and outcomes. Some of the constructs that represent these domains and are considered relevant to the study of teacher education, teaching and the teaching profession are described in Table A1 in the Appendix.

##### Preparatory service

In order to describe the preparatory service, the LAP study gathers information on basic characteristics such as teaching track, perceived learning environment, and different teaching practices applied in lessons given during the preparatory service (see Appendix, Table A2). Questions about all of these aspects were asked in computer-assisted telephone interviews (CATI).

The instrument used for characterising the learning environment focuses on the support dimension of the above-mentioned SSCO model. While most taxonomies of social support distinguish between informational, instrumental, and emotional support (Helgeson, 2003), the LAP study only addresses instrumental support, which has been found to be predictive of emotional exhaustion and self-efficacy (Richter et al., 2011) and “involves people providing concrete assistance” (Helgeson, 2003, 25). However, social support is measured in relation to three important reference groups of trainee teachers: peers, mentor teachers, and the head of the teaching seminar. In addition to social support, the LAP study also gathers information on the challenge dimension of the SSCO model (e.g., constructivist interaction with mentors; see Appendix, Table A2) and the orientation dimension (e.g., integration of theory and practice; see Appendix, Table A2).

To measure instructional practices applied during preparatory service, we used two subscales of the instrument proposed by Weresch-Deperrois et al. (2009). These subscales are informed by the “Standards for teacher education: Educational sciences”, which have been adopted by the *Kultusministerkonferenz* (Standing Conference of the Ministers of Education and Cultural Affairs of the Länder in the Federal Republic of Germany; Kultusministerkonferenz, 2014) and describe relevant instructional skills. However, it turned out that the psychometric properties of the scales were not convincing. Therefore, a different operationalisation was used when surveying the teaching practices of all teachers in computer-assisted web interviews (CAWI; see below).

##### Learning opportunities and professional experiences

In the assessment of teacher competencies, the LAP project placed particular emphasis on competencies related to heterogeneity in the classroom and, in later waves of the survey, on the use of ICT for teaching. Since the development of these competencies depends not least on corresponding learning opportunities, the surveys asked which experiences the respondents had gained with regard to inclusion, multiculturalism, and digital media during teacher training and in the teaching profession (see Appendix, Table A3).

The learning opportunities and professional experiences regarding multiculturalism and inclusion were measured in the online surveys from 2016 onwards, based on instruments proposed by Laschke and König (2014; learning opportunities) and also by the project “Attitudes towards inclusive education in schools” (*Projekt E1NS)* at Hildesheim University (Stiftung Universität Hildesheim, 2016; professional experiences). Starting in 2018, professional experiences with different special education focuses (e.g., visual impairment, autism, or special needs in physical and motor development) were collected in a more differentiated manner, based on an instrument of NEPS Starting Cohorts Grade 5 (SC3) and Grade 9 (SC4) (PAPI 2012/13; LIfBi, 2016). In 2021, learning opportunities and professional experiences related to the use of digital media in the classroom were added to the survey, replacing the focus on inclusion.

As an extension of capturing professional experiences, teachers were asked about the extent to which they perceive a heterogeneous student body as an impediment of their teaching. The dimensions of heterogeneity considered include cultural and social heterogeneity, performance heterogeneity, and differences in behaviour and motivation. The nine-item instrument, based on a measurement by Baumert et al. (2008), has been used in CATI studies since 2020 (see Appendix, Table A3).

##### General aspects of professional competencies

Following the competence model proposed by Baumert and Kunter (2013), the LAP study measures general motivational orientations, beliefs, and self-regulation. General motivational orientations include motivation for choosing teacher education or a career in teaching, teacher enthusiasm, and teacher self-efficacy.

The motivation for career choice was measured twice: The first measurement took place retrospectively in 2014 (wave 8) with regard to choosing a teacher education programme (see Appendix, Table A4; instrument adapted from Pohlmann & Möller, 2010; Retelsdorf & Möller, 2012). Please note that the data is stored in different variables, depending on whether the respondents are still studying for a teaching degree or have already earned a degree in teaching. The variables can and should be analysed together. The second measurement six years later (wave 19) focuses on the career decision of teachers who had not earned a higher education degree in teaching. Part of the instrument is identical to the one used in wave 8, however, some subscales were omitted, the subscale “fallback career” was added (items adapted from Watt et al., 2012), and some items had to be rephrased.

Teacher enthusiasm can be conceptualised as an affective construct that belongs to the domain of positive emotion and intrinsic motivation (Kunter et al., 2011). However, teacher self-efficacy can also be conceived as a specific form of beliefs, namely “self-beliefs” (Kunter & Pohlmann, 2015; Pajares, 1992).

While self-efficacy is classified differently, beliefs about teaching and learning and teachers’ professional self-concept clearly belong to the competence aspect “beliefs”. Beliefs about teaching and learning were measured in all CAWI waves since wave 8, albeit with differing instruments. In wave 8, the German-language questionnaire of “The Teaching and Learning International Survey (TALIS)” was applied (OECD, 2009). Because in this case measurement quality was not good, the LAP project decided to implement a shortened version of the instrument described by Kunter et al. (2017) in subsequent waves. Both instruments cover the dimensions transmission beliefs and constructivist beliefs.

Teachers’ professional self-concept was measured in all CAWI waves from wave 8 onwards, using selected subscales and items of the questionnaire developed by Retelsdorf et al. (2014). Because it became necessary to shorten the survey, the dimension “self-concept in consulting” was omitted from wave 11 onward.

The questionnaire used to measure occupational self-regulation is based on Schaarschmidt and Fischer’s (2001) AVEM inventory. Although this inventory was reduced from eleven dimensions with 66 items to four dimensions with 13 items, the shortened version still showed sufficient measurement quality (Menge & Schaeper, 2019).

##### Specific aspects of professional competencies: dealing with inclusive education, cultural diversity, and digital media; teachers’ stereotypes

Regarding specific aspects of teachers’ professional competencies, the LAP study addresses inclusive education, cultural diversity, and teaching with digital media, and measures corresponding beliefs and self-efficacy expectations (see Appendix, Table A5). While the issue of inclusive education and teaching in culturally diverse classes has been included since CAWI wave 11, teaching with digital media was added to the survey programme later (wave 17 or 19) and partially replaced the topic inclusion.

Information on the quality of the scales used to measure inclusion-related beliefs and self-efficacy expectations can be found in Menge et al. (2021). To date, validity and reliability analyses of the other scales using the LAP data have not been published. However, in NEPS SC3 teachers’ cultural beliefs were measured using an almost identical instrument. The only difference is that in NEPS SC3 the subscale “multicultural beliefs” consists of one additional item (“During counselling sessions with parents who have a different cultural background than I do, I try to respect cultural particularities.”). A recent analysis of this data yielded satisfactory values for Cronbach’s alpha and confirmed the three-dimensional structure of teachers’ cultural beliefs (Schotte et al., 2022).

Stereotypes, conceptualised as “beliefs about the characteristics, attributes, and behaviors of members of a certain group” (Hilton & von Hippel, 1996, 240; cited from Wenz et al., 2016, 3), are a major source of discrimination. Therefore, stereotypes held by teachers about competencies and abilities of different groups may help to explain discriminatory judgements and behaviours and, consequently, educational and performance inequalities (for details see Wenz, 2020; Wenz et al., 2016). To measure teachers’ stereotypes, Wenz and colleagues (Wenz, 2020; Wenz et al., 2016) developed a questionnaire that was first used in NEPS Starting Cohort “Kindergarten” (SC2). This questionnaire assessed teachers’ stereotypes regarding reading and mathematical competencies in the following groups: female and male students; students with low, middle, and high socioeconomic backgrounds; and students of Turkish and Russian origin, immigrants in general, and ethnic majority students in general. A slightly different instrument was implemented in the LAP study (see Appendix, Table A5). While focusing on the same social groups, the 18 items address (prospective) teachers’ stereotypes relating to reading competencies and parental support.

##### Contextual information on the professional situation

As important contextual information on employment as a teacher (see Appendix, Table A6), we measured the type of school, the (level and size of) classes and the subject groups that are (predominantly) taught by the respondents. This contextual information plays an important role especially when corresponding differentiations are required in data analyses.

Since dealing with heterogeneity in the classroom is one of the focal points of the LAP project, we also collected information on the proportion of students in the school with a migration background and the composition of students in the classes taught.

In order to be able to analyse, for example, the influence of the teaching staff, the school management or the classroom context on teachers, the length of time the participants had been working at their school was also recorded. As a supplement to the questions about the leadership style of the school management (see below and Appendix, Table A6), the participants were also asked if they worked as (deputy) head teachers, since in this case they were not asked the further questions on leadership style that would require them to evaluate themselves.

##### Teaching practices

It goes without saying that students’ competence development depends on the way teachers teach and the quality of instruction (Hattie, 2009). Classroom management (**S**tructure), cognitive activation (**C**hallenge), and constructive support (**S**upport) are considered to be central dimensions of teaching quality (Klieme et al., 2006). Together with **O**rientation as a fourth dimension (Radisch et al., 2007) these factors are the components of the above-mentioned SSCO model.

Cognitive activation was initially measured using three items adapted from Kunter et al. (2017) (see Appendix, Table A7). To increase the internal consistency of the scale, two more items from the same source were added as of wave 14.

As has often been the case in previous research, the LAP study distinguished two dimensions of classroom management: disruptions/effective use of time and monitoring. Initially, both dimensions were measured using three items from Kunter et al. (2017). Due to the low Cronbach’s alpha value of the monitoring scale, two additional items from other instruments were later introduced.

Central features of the support dimension are positive emotional relationships, support for autonomy and competence, and social embedding (Bäumer et al., 2019). Measures of support also include internal differentiation and individualisation of instruction (Kunter et al., 2013). Differentiation and individualisation, in turn, are crucial for the participation of all students in the classroom, i.e., inclusion (Gebhardt et al., 2014). These teaching principles are not only characteristic of inclusive teaching but also of a supportive approach to dealing with cultural heterogeneity. Just as for the two previously mentioned facets of teaching practices, differentiation/individualisation was measured four times, in CAWI waves 11, 14, 17, and 19.

The next-to-last instrument listed in Table A7 in the Appendix has a somewhat different focus. It is not directed towards teaching practices but rather towards the emphasis teachers give to developing different ICT-based abilities. The items were taken from Vennemann et al. (2021) and applied once in CAWI wave 19.

##### Proactive behaviour in occupations

In 2020, the LIfBi launched the first open “Call for Modules” (CfM), giving external researchers the opportunity to incorporate additional questions in the NEPS survey programme. Three different proposals were selected and one of them was chosen to be implemented in the NEPS SC5 survey 2022. The proposal “The innovative teacher? Proactive behaviour in different occupations” includes a five-item scale (see Appendix, Table A7) measuring proactive professional behaviour, and was submitted by Mareike Kunter, Franziska Baier, Julia Dohrmann and Verena Jörg from the Leibniz Institute for Research and Information in Education (*Leibniz-Institut für Bildungsforschung und Bildungsinformation* (DIPF)). Proactive behaviour, defined as self-initialised and change-oriented activities (Hüttges & Fay, 2019; Ohly et al., 2006; Thomas et al., 2010), is a prerequisite for dealing both with changes in society and in the school system, and has been shown to be predictive of occupational success (Hüttges & Fay, 2019; Thomas et al., 2010). It was measured using the subscale “Voice”, which is conceptualised as the “individuals’ propensity to proactively discuss change-oriented and constructive ideas” (Thomas et al., 2010, 277). The operationalisation is based on the instrument of van Dyne and LePine (1998), which has been translated, shortened and adapted for teachers by Kunter et al. (2017) (see Appendix, Table A7). To be able to compare proactive professional behaviour in different professions, two versions exist; a version for teachers, taken from Kunter et al. (2017), and a slightly modified version for study participants in other professions.

##### Experiences and situation during the Corona pandemic

As already mentioned in various publications (e.g., Fickermann & Edelstein, 2021), the Covid-19 pandemic and school closures required many short-notice changes to regular approaches to teaching in schools very early on, thus exposing students and their families—but also schools and teachers—to manifold challenges and difficulties. Various aspects of these new requirements and adaption to teaching were conceptualised and measured during and after the pandemic-related school closures (see Appendix, Table A8). (Prospective) teachers were asked first and foremost about the situation in their schools and classes and—if they were still studying or in preparatory service—how much the pandemic affected the course of study or preparatory service or the study situation (e.g., taking exams, attending courses).

Additionally, data was collected on the challenges faced by teachers during school closures (e.g., with respect to providing learning material, motivating students for remote learning, support by colleagues and principles). Other questions refer to communication with families and provision of information and learning materials for students during school closures (e.g., online platform, school clouds, telephone or regular mail). The scales were adopted and partly adapted from similar research projects on teaching during the pandemic-related school closures (e.g., NEPS-C (https://www.neps-data.de/Data-Center/Data-and-Documentation/NEPS-C); Lorenz et al., 2020).

As mentioned above, teachers were asked about their attitudes towards digital media in schools, their self-efficacy with respect to using digital media for teaching (see Appendix, Table A5), and teaching with digital media both in general (see Appendix, Table A3) and specifically with regard to school closures. The corresponding questions, adapted from Bos et al. (2010), measure the extent to which the use of digital media for various teaching purposes changed after the reopening of the schools compared to the time before the schools were closed.

Pandemic-related questions were also asked to all study participants of NEPS SC5. The first survey with pandemic-related questions was conducted in May 2020 during the first nationwide school closures in Germany. All panel members were invited to participate in a web-based survey with general questions regarding their family situation and child-care arrangements in the context of closed education institutions and—if they had children themselves at that time—how well they managed at home with remote schooling. Furthermore, they were asked about their own experiences with remote learning if they still attended formal education as well as about life satisfaction, and their work or study situation during the lockdown. In the following surveys in autumn 2020 through to spring 2021 as well as one year later repeated measures of pandemic-related instruments were included in the regular questionnaire programme.

##### Professional development activities

Lifelong learning is embedded in educational and professional careers (Allmendinger et al., 2019). Therefore, data on continuing education is also collected in the NEPS SC5 life course interviews. As continuing education plays an important role in maintaining and expanding teachers’ professional competence (see Chapter 1), additional questions have been included in the surveys since 2019 (see Appendix, Table A9).

All teachers who reported at least one further training in the life course interview were asked whether they had participated in PD activities related to their job as a teacher in the last twelve months. If yes, the respondents were asked to indicate the topic(s) of the training. Ten of the eleven topics presented were selected from TALIS (Teaching and Learning International Survey) 2008, conducted by the German Education Union (GEW) (Gagarina & Saldern, 2010), TALIS 2013 (Europäische Kommission/EACEA/Eurydice, 2015), IGLU (International Primary School Reading Survey) 2016 (Hußmann et al., 2017), and the general teacher questionnaire of NEPS SC4. Where necessary, the original items were modified; one item was developed in-house. The selection covers the subject-specific/content pedagogical, general pedagogical and method-oriented topics identified by Richter et al. (2013).

In addition, data was collected on factors that influence teachers’ participation in PD activities. Referring to Richter et al. (2010), who used Cookson’s (1986) model for explaining participation in continuing education and applied it to the situation of teachers, the LAP project distinguishes between context-specific, social, psychological, and situational factors.

Apart from predictors described in the previous sections, the LAP, therefore, measured teachers’ beliefs regarding the importance of continuing education and the relevant school context. The subjective importance of continuing education was operationalised using the scale from the IQB National Assessment Study 2011 (Richter et al., 2014), which was only slightly modified. Regarding aspects of the relevant school context, the focus was placed on attitudes towards continuing education in the teaching staff and in the school, support from school principals (the aforementioned aspects were measured with selected items from the IQB National Assessment Study 2011), and on the availability of resources for PD activities (measured by adapted items of IGLU 2001 (Bos et al., 2005)) (see Appendix, Table A9).

##### Teacher cooperation and school leadership styles

As pointed out in Chapter 1, colleagues and school leaders are important context factors, predictive of various other factors at the teacher, student, and school levels. Several models have been proposed in order to conceptualise and describe cooperation among teachers. The LAP study opted for the three-stage model of Gräsel et al. (2006), which is strongly influenced by the work of Little (1990), and her typology of four ideal typical forms of teacher cooperation. Gräsel et al. (2006) distinguish three dimensions or stages—exchange, joint work, and co-construction—which are characterised by increasing requirements. The three dimensions were measured in CAWI waves 14, 17, and 19, each with three to four items (see Appendix, Table A10).

There are several models for conceptualising leadership and classifying leadership styles (overview in Bush & Glover, 2014; Greubel, 2017). In accordance with the relevance of instructional and transformational leadership for teachers, students, and schools (see Chapter 1), the LAP study also includes scales that measure these leadership styles of school principals, as perceived by the teachers. The instructional leadership scale, taken from Pietsch et al. (2014) and slightly modified, initially consisted of four items and was later expanded to five items. The transformational leadership scale is based on the German adaptation (Heinitz & Rowold, 2007) of the Transformational Leadership Inventory (TLI) by Podsakoff et al. (1990). Of the six dimensions that measure transformational leadership, three subscales were selected (articulating a vision, fostering the acceptance of group goals, providing an appropriate model), each comprising three items. The exact wording was taken from Ewen (2013), who adapted the questionnaire to the school context. These instruments have also been used three times since CAWI wave 14.

##### Occupational well-being

Indicators of occupational well-being are outcomes of educational and professional careers, situations, and experiences, and can therefore be considered as non-monetary returns to education (Gross et al., 2019). On the other hand, they are predictors of educational and occupational decisions, competencies and behaviours and can help to explain dropping out from preparatory service and from the teaching profession (Blömeke et al., 2017; Klassen & Chiu, 2011; Skaalvik & Skaalvik, 2011, 2017), instructional quality (Klusmann et al., 2008; Kunter et al., 2013), and student outcomes (Arens & Morin, 2016; Klusmann et al., 2016; Klusmann & Richter, 2014).

The LAP study focuses on two aspects of occupational well-being: emotional exhaustion and job (or career) satisfaction. Emotional exhaustion “represents the basic individual stress dimension of burnout […] and refers to feelings of being overextended and depleted of one’s emotional and physical resources” (Maslach et al., 2001, 399). It was operationalised using the slightly modified four-item scale proposed by Kunter et al. (2017) (see Appendix, Table A11). This scale in turn is based on the Maslach Burnout Inventory (MBI; Maslach & Jackson, 1981) and the German translation by Enzmann and Kleiber (1989). In the LAP, the four items were presented once for measuring emotional exhaustion during the preparatory service and annually from CATI wave 10 onwards for target persons working as teachers.

Job satisfaction can be defined as “a positive (or negative) evaluative judgment one makes about one’s job or job situation” (Weiss, 2002, 175). Accordingly, Skaalvik and Skaalvik (2011, 1030) conceptualise teacher job satisfaction as teachers’ affective reactions to their work or to their teaching role. Some researchers, however, distinguish between career satisfaction (*Berufszufriedenheit)* and job satisfaction (*Arbeitszufriedenheit)* (e.g., Abele et al., 2011; Hagmaier et al., 2018): While job satisfaction refers to the evaluation of one’s working conditions (Abele et al., 2011), career satisfaction is considered a broader construct that takes “a long-term perspective to work experiences” (Hagmaier et al., 2018, 142) and refers to the “evaluation of the accumulated experiences in one’s career” (Hagmaier et al., 2018, 142). Focusing on the overall assessment of career choice, the four-item scale used in the LAP study and taken from Kunter et al. (2017) represents career satisfaction rather than job satisfaction. Data was collected from respondents who are in or have completed preparatory service or who are working as teachers.

### 2.6 Quality Control

#### Study planning in the larger NEPS context

In the NEPS, a large network of education researchers from different disciplines and institutions works together and coordinates and exchanges information on the survey and testing programme. A master plan meeting, several coordination meetings, and a meeting for the final approval of the survey or test are held for each study. To ensure high data quality and comparability across cohorts and over time, several standardised processes for the development of survey instruments and fieldwork have been implemented in the NEPS. These processes have also been adopted by the LAP study.

#### Selection of survey content and assurance of the quality of the constructs

Once relevant contents for the survey had been chosen based on extensive literature reviews and pre-announced in the consortium, potential measures were examined. In most cases, the LAP had to rely on tested and well-established operationalisations and could not develop and pre-test new instruments. The quality of established questionnaires was controlled by reviewing the documentation and further literature regarding survey instruments and theoretical approaches, contacting item developers, and performing secondary analyses using available data from other studies in which the instruments were used.

In many cases, time restrictions made it necessary to shorten the original measures for an application in the LAP programme. In order to obtain a short scale with the best possible scale properties, statistical criteria such as reliabilities, internal consistency, discriminant and convergent validity, discriminatory power of items, and factor structure with and without certain items were all considered in addition to theoretical considerations and occasional recommendations from experts.

After the initial use of the instrument in the LAP study, each instrument was checked regarding, for example, factor structure, internal consistency and item non-response. In individual cases, if the scale properties did not meet the quality standards, the instruments were modified for the next use. Following the maxim “If you want to measure change, don’t change the measure” (Beaton & Zwick, 1990, cited from Beaton & Barone, 2017, 252), moderate adjustments to the wording or response scale were made rarely and only when necessary, e.g., to take account of changing conditions regarding attainable degrees or childcare arrangements. Before any adjustments were made, the measurement accuracy and comparability were weighed up for each individual case. Adjustments are documented and can be recognised in the data by the versioning of the variables concerned. For quick access to information, a documentation on the LAP survey instruments, although not available at time of writing, will be published in the near future, describing the properties, sources and changes in the instruments.

#### Questionnaire construction, field preparation and field control

The NEPS/LAP studies were mainly conducted by the survey institute infas (infas Institute for Applied Social Sciences, Bonn, Germany).^3^ To ensure high survey quality, several steps were taken, which partly differ between online and telephone surveys.

For online and computer-assisted telephone surveys, the survey institute and members of the LAP project and the NEPS consortium thoroughly controlled the programming in several test loops. The surveys were checked for correct filtering, proper construction of auxiliary variables and – in the case of web-based surveys – correct display on different technical devices (e.g., PCs, laptops, smartphones). Since 2016, the online surveys have been optimised for different devices. When programming was completed, an additional data storage check was performed.

In the case of computer-assisted telephone surveys, interviewers were trained before each new survey. The interviewer training was complemented by an interviewer manual that provided important information about the sample, the survey programme, and the interview process. Supervisors in the CATI call centre and a field operations manager were available for support.

Once a study was in the field, the survey institute regularly reported the number of contacted target persons, realised interviews, and interview duration. At several points during the field period, all data collected up to that point was transmitted. This intermediate data was checked in detail. This allowed for accurate field control and timely intervention in case of any anomalies. To control the quality in the field, several telephone interviews were recorded and examined. Additionally, feedback from interviewers in telephone surveys and comments from target persons in online surveys were analysed and checked for potential adjustments for the following studies.

### 2.7 Data anonymisation and ethical issues

To participate in the panel study, students had to give prior informed written consent. Moreover, informed consent to take part in the study was also given by the educational institutions, so their students were allowed to be contacted. Data protection and security officers of the LIfBi and DZHW approved the consent procedure.

The anonymity of the participating students and the respective HEIs is guaranteed by following two principles of the NEPS anonymisation concept: First, disclosure of participants’ identities should be impossible and, second, a high utility of the data should be preserved (Schier et al., 2019). Therefore, information that would allow identification of individuals or HEIs (e.g., names, addresses) is not provided in the Scientific Use Files. Direct identifiers are separated from the data and are not delivered from the survey institute to the LIfBi. In addition, the anonymous identifiers used by the survey institute are replaced by new ones in the SUFs.

To provide secure and comfortable data access for the scientific community, a combination of five approaches has been implemented (see Schier et al., 2019).

*Organisational data protection:* The data is only available to the scientific community. Access to the data is managed by the LIfBi Research Data Center, which checks the status of the data user, their connection to a university or a research institute and the scientific interest of the data request.

*Legal data protection:* Data users are informed about data protection and data security. They are committed to data protection and must sign a contract with corresponding regulations.

*Statistical data protection:* Techniques of statistical data protection are used to ensure the respondents’ privacy by generating factually anonymous data. Various modification approaches exist and are applied in the NEPS, e.g., aggregating data or slightly changing variable values. Which approach is chosen primarily depends on the way in which data access is realised (see below).

*Informational data protection:* LIfBi staff offer a special training programme to data users that explains the complex data structure and gives information on data protection and data security. Furthermore, detailed documentation is provided that includes information about data protection and anonymisation measures.

*Technical data protection:* As the data collected in LAP/NEPS is digital, technical data protection in the form of hardware and software solutions is essential. One area of technical data protection concerns data dissemination. Depending on the sensitivity of the data, LIfBi offers three different modes of access to the Scientific Use Files (see Chapter 3.7). Regardless of the access mode, all data is anonymised using random person and institution identifiers so that neither individual study participants nor HEIs can be identified. Information that is more sensitive is only available under restricted conditions following data protection rules. For example, information on federal states and places of residence of participants is not available in the downloadable dataset but in a data-secured environment that can only be accessed with an additional data usage contract (Fuß & Wenzig, 2019). The anonymisation manual on neps-data.de provides information on which variables are available for which access mode (see https://www.neps-data.de/Data-Center/Data-and-Documentation/Start-Cohort-Students/Documentation).

### 2.8 Existing use of data

All publications that make use of the NEPS data can be found on the LIfBi website https://www.neps-data.de/Project-Overview/Publications. In December 2022, 140 publications could be counted for the cohort of first-year students, of which 19 relate to (prospective) teachers; they are listed in Table A12 in the Appendix. In addition, another paper has been accepted and will be published in 2023. Based on the Data Use Agreements concluded with the LIfBi for accessing the NEPS and LAP data (see https://www.neps-data.de/Data-Center/Research-Projects), the database is used by a large number of research projects. In total, there are 4,446 contracts for all NEPS Starting Cohorts combined. Consequently, more research results will be published shortly.

## 3 Dataset description, access and how to use the data

### 3.1 Repository location

All data collected by the LAP project is fully integrated in the datasets of NEPS SC5, which are published as Scientific Use Files by the LIfBi Research Data Center.

The most recently released SUF of NEPS SC5 and LAP data is version 17.0.0 (doi:10.5157/NEPS:SC5:17.0.0; NEPS Network, 2022). Data users are recommended always to use the most recently published issue. Older versions and their accompanying materials remain accessible.

After having signed the mandatory Data Use Agreement (see also Chapter 3.7), the datasets can be downloaded from the website https://www.neps-data.de/Data-Center/Data-and-Documentation/NEPS-Data-Portfolio. More information on how to access the data can be found at: https://www.neps-data.de/Data-Center/Data-Access.

### 3.2 Object/file name

Due to the complex structure and the rich amount of NEPS/LAP data, the data is stored in not just one but several different datasets (see Table 3). To help data users with this complexity, the LIfBi Research Data Center provides a merging matrix which gives an overview on how to link information from different datasets. In addition, users may find the semantic data-structure file useful. This file does not contain actual data but meta-data such as variable names, labels and scheme options. With this information data users can explore the data structure without signing a Data Use Agreement. For more information on the data structure and its complexity, see the data manual. All the materials mentioned are listed in Table 4.

### 3.3 Data types

The NEPS SC5/LAP Scientific Use File contains different types of data but mostly primary data from the target persons. Information on other (context) people such as partners and children was collected from the participants themselves. These data files include many derived variables, for example, classifications of occupations (e.g., ISCO, EGP) and education (e.g., ISCED, CASMIN), aggregated test scores and plausible values, and auxiliary variables.

In addition to primary data, methods data, context data, and process-generated employment-related social welfare data (administrative data) is also available. Context and administrative data can only be analysed on-site at the LIfBi and, in the case of the last-mentioned data, at the Institute for Employment Research (IAB) in Nürnberg or at some of the other IAB locations. In the methods data files users find paradata such as occurrence of problems during interviewing or testing, length of interview or survey, participation, and, in the case of telephone interviews and competence tests, information about the interviewer.

For NEPS SC5/LAP two types of context data are provided: Information for all 413 higher education institutions listed in the codebook of the Federal Statistical Office in 2010/2011, and information on the study programmes (aggregated to subject areas) offered by these HEIs (see Weber, 2014). Examples of variables to be found in this dataset are size and institutional control of HEI, gender composition and financial resources for each subject area in a HEI, and economic and social structure of the regional context of a HEI. The second type of context data provides information on the area of the respondents’ home such as age distribution, house type, milieus, family structure, and probability of payment default (see Schönberger & Koberg, 2017).

The last dataset to be mentioned is the NEPS-SC5-ADIAB (doi:10.5164/IAB.FDZD.2112.en.v1; Bachbauer et al., 2022). It is provided jointly by LIfBi and IAB and consists of the NEPS SC5/LAP data and the administrative data of the IAB. This administrative data includes, for example, information on employment history, benefit recipient history, and jobseeker history.

### 3.4 Format names and versions

The download version of the SUF is provided as Stata and SPSS files and data users need their own software licences. If users analyse the data via remote access (RemoteNEPS) or on site, they use the statistical software implemented on the LIfBi server system. In this case, Stata, SPSS, and R are available but not, for example, Mplus.

### 3.5 Language

Most of the documentation and supplementary materials are provided in English. The Stata files contain German and English variable and value labels. Stata users can easily choose between the languages (de, en) with the Stata command “label language *languagename*”.

### 3.6 License

All NEPS/LAP data published as SUFs is only accessible for scientific use by researchers, due to German data protection laws and corresponding participation agreements with all survey participants. As data access is regulated by Data Use Agreements, the application of (open) licenses is not required.

### 3.7 Limits to sharing

The LIfBi Research Data Center aims to make the NEPS/LAP data available to researchers as quickly as possible and tries to publish the Scientific Use File within 18 months of the end of fieldwork. Data access is granted to the scientific community for scientific purposes only and on condition that a Data Use Agreement is concluded (see below and Fuß & Wenzig, 2019). The amount of sensitive information that researchers can analyse depends on the access mode: The downloadable SUF version is characterised by the highest degree of data modification and anonymisation and contains little sensitive information. Datasets available via remote access provide more sensitive information than the download SUF. Finally, the on-site version, which requires a guest stay at the LIfBi, has the lowest level of anonymisation and the highest degree of sensitivity. If data users want to analyse the remote access SUF data or the on-site data version, they need to sign supplements to the NEPS Data Use Agreement. Using the data via remote access also requires a biometric authentication (keystroke biometrics). Further information on which variables may be restricted in use due to sensitivity and are therefore only accessible via remote access or on site can be found in the anonymisation report, which is released with every data issue (https://www.neps-data.de/Data-Center/Data-and-Documentation/Start-Cohort-Students/Documentation; short overview: https://www.neps-data.de/Data-Center/Data-Access/Sensitive-Information).

### 3.8 Publication date

The most recently published Scientific Use File has been available since 7 November 2022. With each new version the data will be extended and updated (e.g., by adding additional survey waves, data sets or corrections). Information on the publication date of earlier SUF releases can be found online (https://www.neps-data.de/Data-Center/Data-and-Documentation/Start-Cohort-Students/Data-and-Citation). By clicking on the respective link in the table, the corresponding documentation is accessible.

Since the last NEPS SC5/LAP survey was conducted in 2022, there are only a few panel waves that have not yet been published. According to the publication guideline mentioned above, publication of the 2021 CATI can be expected around spring 2023 (SUF version 18) and the release of the combined online and telephone survey conducted in 2022 is due in spring 2024 (SUF version 19). The data release schedule on the NEPS website (https://www.neps-data.de/Data-Center/Overview-and-Assistance/Zeitplan-en-US) is constantly updated.

### 3.9 FAIR data/Codebook

*Findability:* LAP data is integrated in the NEPS SC5 datasets and is therefore easy to find via the DOI (digital object identifier). Each subsequent data release is given its own and thus unique DOI. Part of this identifier code is the release number of that Scientific Use File, which is also used to link all related materials to a specific issue. In this way, data users can easily connect documentation material such as the data manual or codebook to the specific data. The LIfBi Research Data Center provides a wealth of information and documentation materials on their website (https://www.neps-data.de/Data-Center/Data-and-Documentation/Start-Cohort-Students/Documentation).

*Accessibility:* Access to the data is free of charge and managed by the LIfBi Research Data Center. Each researcher who wants to use the data has to sign a Data Use Agreement (see Chapter 3.7 for more information). It is recommended for new users to participate in one of the NEPS data training courses, which take place on a regular basis and are offered free of charge by the LIfBi Research Data Center. A signed Data Use Agreement is not limited to a specific SUF release; if a new SUF version is released during the contract period, data users are free to use the new issue. Information connected to outdated releases is still accessible via the LIfBi Research Data Center website.

*Interoperability:* Interoperability is ensured by providing SUFs in widely-used data formats (e.g., Stata or SPSS). For more information on how to access the NEPS/LAP data see Chapter 3.7.

*Reuse:* The LIfBi Research Data Center provides rich documentation materials (like codebooks, questionnaires and data manuals) and help for data users (like NEPSplorer, NEPSforum, data training courses). The website lists a huge variety of open access publications (e.g., NEPS Survey Papers, LIfBi Working Papers) and moreover, each data usage contract is listed with a short description on the website, which gives an opportunity to view ongoing research. The Data Use Agreement necessary to use the NEPS and therefore the LAP data clearly defines the regulations that all data users must follow.

*Relevant meta-data***:** In addition to those materials mentioned in chapter 2.5.1, there are some further helpful sources of information**:** methods datasets; samples and weights; via NEPSplorer: information on what items were part of which survey(s), to what theoretical construct they belong, and which references have to be cited (BibTeX); and soon: documentation of the LAP survey instruments.

### 3.10 How to analyse the LAP data – technical advice, special variables and more

Due to the complexity of the study design and the longitudinal information on the teacher education students, working with the data can be a challenge. In particular, identifying the LAP target persons such as teacher education students, future teachers in preparatory service and in-service teachers can be difficult since no separate dataset for LAP exists, but rather all information is integrated in the NEPS SC5 SUF.

To facilitate data use, specific variables can be used to identify LAP target persons and the phase of teacher education they were in at the time of (selected) surveys.

#### Identifying teacher education students in NEPS SC5

Information about the first study programme of the study participants was collected in two different surveys, the initial paper-and-pencil questionnaire and the first telephone interview. Respondents who classify themselves as teacher education students in the initial PAPI can be identified with variable tg02001 in the data file ‘pTargetCATI’, which distinguishes between various degrees, or variable tg02001_g1 (also in ‘pTargetCATI’), which takes the possibility of polyvalent bachelor’s degrees with the option of specialising in teacher education into account. To identify teacher education students at the beginning of their studies in the winter term of 2010/2011 with data from the CATI, variable tg24201_g1 in ‘pTargetCATI’ can be used. Since the information on the intended degree at the start of studies was gathered in different ways, the number of teacher education students differs between the first PAPI and CATI.

Whether or not study participants were enrolled in a teaching degree programme in the course of the subsequent educational history can be determined from the episode data in the data file ‘spVocTrain’ using the variable ts15221_g1 (Intended vocational qualification, revised) in combination with tg24201 (Intended teaching degree). A more detailed description of the different ways to identify teacher education students can be found in the most recent data manual for the Scientific Use File of NEPS SC5.

There is more than one possible answer to the question of which respondents in CAWI waves 2, 4, 6, and 8 were enrolled in a teaching degree programme at the time of the survey, but regardless of the solution, data users need to combine information from the data file ‘spVocTrain’ of the last CATI in which the respondents participated with information from the respective CAWI.

As of wave 11, auxiliary variables have been introduced to help identify LAP target persons at the time of the survey and to distinguish between different phases of teacher training and employment. The variables tg60012 and tg60017 refer to CAWI waves and are stored in the data file ‘pTargetCAWI’. Variable tg60017 is an updated version of tg60012, which was introduced in wave 14 and considers whether respondents denied a previously stated teacher (education) context later in the survey. Similarly, variable tg60013 refers to CATI waves. These variables exist in different versions (see the most recent data manual) and capture the training/employment status of (future) teachers at the time of the telephone or online interview.

#### Coding and identifying the type of (intended) teaching degree

Information on the type of intended teaching degree was collected with an open-ended question and then coded based on a classification of the Standing Conference of the Ministers of Education and Cultural Affairs of the Länder in the Federal Republic of Germany (*Kultusministerkonferenz*/KMK). The coding scheme, however, does not distinguish teacher education programmes that include several levels. Instead, the answer was coded under the highest level, when more than one type of teaching degree or degree programmes spanning several levels were reported.

Information on the type of intended teaching degree that refers to the first study programme in the winter term of 2010/2011 can be found in ‘pTargetCATI’ (tg24202_g2, tg24202_ha, tg03001_g2). The intended teaching degree reported for different study episodes is stored in ‘spVocTrain’ in variable tg24202_g1. Determining the type of teaching degree (and subjects) in the CAWI waves is somewhat more complicated, as the information was not newly collected but was rather updated when respondents had changed their intended degree (or subject). Therefore, it is necessary to use information stored in ‘spVocTrain’ and perhaps combine it with data from the CAWI.

#### Identifying teacher candidates in preparatory service

Information on whether participants attend preparatory service is provided in ‘spEmp’. For each employment episode (including working as trainee teacher), (1) the type of employment (e.g., employment with training character, self-employment) is determined, (2) if applicable, the type of employment with training character is recorded (variable ts23214), and (3) in case of a *Referendariat* (preparatory service), it is asked whether the *Referendariat* qualifies for teaching. This information is stored in tg64001 (Teaching Referendariat yes/no).

From wave 11 onwards, the auxiliary variables (and their versions) tg60012 or tg60017 in ‘pTargetCAWI’ and tg60013 in ‘pTargetCATI’ can be used to identify participants who are in preparatory service at the time of the survey.

#### Identifying in-service teachers

The information on whether the study participants are already working as teachers at the time of the interview is again provided in the auxiliary variables from wave 11 or wave 14 onwards. If data users are interested in whether panel members have worked as teachers throughout their employment history, they can use the information on occupations in the episode dataset ‘spEmp’. This information is collected with an open-ended question and then coded using established classifications of job titles. For example, in the German Classification of Occupations of the Federal Employment Agency (KldB 2010; variable ts23201_g2) all codes beginning with 841 stand for teachers in general education schools, and codes beginning with 842 refer to those teaching vocational subjects, in-company training and in-company pedagogy. Depending on the specific research interest and questions, other classifications may also be used.

Please note: Variables based on job titles do not distinguish between trainee teachers and in-service teachers. A primary school teacher in the preparatory service receives the same code as a fully qualified primary school teacher. To make this distinction, the variable tg64001 can be used.

#### Auxiliary variables that data users shall use with caution

The auxiliary variables tg60011 (wave 8), tg60014 (wave 9), tg60015 (wave 10), and tg60016 (from wave 13 on) have been generated for purposes of navigation through the survey only. Therefore, it is highly recommended to use these variables with caution. While they may give a first overview of the data, for detailed analyses the original episode data should be used.

#### Missing data and weights

Different codes distinguish between the types of missing values that appear in the data. All missing values are negative or defined as “system missing”. Basically, three categories of missing codes are distinguished: First, *item non-response* if a study participant did not (validly) answer the question. Second, *not applicable* including missing values that occur when an item does not apply to a respondent. And third, *edition missings*, which are generated in the data preparation process and include codes for anonymised data (see the Data Manual for more information).

Regarding unit non-response in the individual surveys and to account for the sampling procedure, weights are provided for each wave. Detailed information on the construction of design and panel weights, as well as on their successive adjustments, is given by Zinn et al. (2017). Weights for the latest wave (17) are described in Ziesmer (2022); they will be updated for the upcoming waves.

#### General recommendations

Versioned variables: Variables sometimes need to be modified (e.g., because of additional categories or corrections); such changes result in different versions of the variables. All versioned variables can be identified by the suffix “_v*” added to the variable name. All those changes are documented in the release notes of each published Scientific Use File and the data manual. Additional information can be retrieved with the NEPS-specific Stata command “infoquery *varname*”.^4^

Using spell data/harmonised episodes: If users want to analyse information stored in spell data format, they are advised to read carefully the corresponding chapters in the data manual. In particular the use of subspells and harmonised episodes may be challenging.

Handling participation or dropout status of respondents: In all NEPS SC5 surveys a special definition of participation status was applied. It differentiates between respondents who participated in the previous CATI or who are considered as temporary dropouts. Temporary dropouts are defined as panel members who did not participate in the last or the last two CATIs. Therefore, the gross sample of each survey consists of panel members who took part in at least one of the last three CATIs (and who did not withdraw their consent prior to the following survey commencement). If participants did not take part in any of the last three telephone surveys, she or he is defined as final dropout. These differentiations are only made for telephone surveys. The gross sample of an online survey is therefore nearly identical to that of the following CATI. For all participants, participation status (at the time of each interview) is stored in the variable tx80220 in the data file ‘CohortProfile’.

Further helpful tips and advice for working with NEPS data in general, can be found in the chapters by Bela (2016) and Fuß and Wenzig (2019).

## 4 Reuse potential

### Strengths and limitations of the data and data collection process

The LAP project has created an important and unique database on (prospective) teachers that has significantly expanded the existing data for empirical educational research in Germany. Combined with the NEPS study, it provides longitudinal information from over ten years of panel surveys, covering the entire teacher education phase and early years in the teaching profession with a large sample of (prospective) teachers in Germany. Unlike other large teacher surveys in Germany, the LAP data is not limited to certain German federal states, school types or subjects taught. Consequently, they allow for a more comprehensive analysis of teacher training and the teaching profession as well as comparisons between different teaching programmes. Through the publication of the Scientific Use Files (currently SUF 17-0-0), the data has the potential of being used for a variety of (secondary) analyses regarding relevant educational processes within the different phases of teacher education and aspects of professional activities of teachers.

Not only does the data provide the empirical basis for numerous questions on teacher education, the transition to the teaching profession, and the first years in the teaching profession, but furthermore the measurement instruments (further) developed in the project are also beneficial for future research on these issues. In order to cover a variety of relevant topics without overburdening respondents, many short scales were developed based on existing instruments. Sometimes, the use of short instruments is viewed critically; however, their quality was examined in detail and is therefore ensured. Documentation on the items and scales used in the LAP study is being prepared and will be published in the near future.

As is common in longitudinal studies, the NEPS/LAP study is confronted with significant panel attrition. Due to the wide range of personal information about the respondents, their psychological characteristics and life circumstances, interesting methodological analyses of panel attrition are possible.

### Research potential

The data can be used to analyse the educational and occupational trajectories as well as the professional situation, professional practices, and self-assessed competencies of (prospective) teachers. A wide range of research questions about teacher education can be answered, such as the factors that influence educational decisions and trajectories, educational outcomes, and the importance of learning environments for competence development and educational decisions.

Regarding educational choices, little is known about the decision to stay in teacher education, to move to non-teaching programmes, to drop out of higher education or to move from non-teaching programmes into teacher education. Furthermore, moves within teacher education, e.g., changing the teacher training track or the teaching subject, have rarely been examined. Because the NEPS collects detailed event history data on educational biography, including moves to other higher education institutions, it provides a unique opportunity to examine the course of studies of teacher education students.

Regarding the teaching profession, for example, it is possible to examine the probation in the teaching profession or the influence of educational experiences in higher education and preparatory service on professional practices. The data also allows for analysing in-post learning, as the third phase of teacher education in Germany. In this context, it is possible, for example, to study the determinants of participation in further education or to investigate how collegial cooperation, the leadership behaviour of principals, and the participation in continuing professional education influence the professional competencies, well-being, resilience, emotional exhaustion, and career retention of teachers at the beginning of their career.

The sample also includes participants who chose teaching as a second career and did not pursue a traditional teaching degree. The data makes it possible to examine the factors that lead to the career decision of these teachers and to compare their career choice motives and self-assessed competencies with their traditionally certified colleagues.

By linking the LAP data with NEPS data, it is also possible to address a wide range of further research questions, including comparisons between different career choices and professions. For example, one can compare the professional well-being between teachers, lawyers, physicians and other occupational groups in order to check whether teachers represent a particularly stressed occupational group. One can examine how the choice for the teaching career or other career paths is influenced by interests, personality traits and school performance. It can also be analysed as to whether students from different disciplines differ in terms of study duration and dropout risk, or whether and to what extent the transition to the labour market after graduation and salaries depend on the field of study.

### Implications for practice and policy

LAP data can be used to reduce research gaps in empirical teacher education research and expand knowledge in the field of teacher-related research. From the project’s research findings published and presented at conferences so far, as well as from further research with the data, recommendations can be derived for practice (e.g., the design of teacher education) as well as for education policy and monitoring. The findings may also provide the basis for reform measures in teacher education. For example, studies on predictors of dropout and career change can help to investigate some of the causes of the (internationally prevailing) shortage of teachers and to derive recommendations on how to support trainee teachers in successfully completing their teacher training and to make the teaching profession more attractive again. And by examining second career teacher more closely it will be possible to better assess their potential and risks and identify their support needs, regarding, for example, the transition into the teaching profession and their teaching quality.

## Additional File

The additional file for this article can be found as follows:

10.5334/jopd.76.s1 Appendix. Tables A1 to A12.

## Footnotes

In the following, we refer to this starting cohort as NEPS SC5.

The Corona pandemic was addressed for a second time in the autumn 2020 web survey of NEPS SC5.

Only the web-based surveys of waves 2, 4, 6, and 8 were administered by the DZHW.

This Stata command is part of the package ‘NEPStools’ that can be found at: https://www.neps-data.de/Data-Center/Overview-and-Assistance/Stata-Tools.

## References

[1] Abele, A. E., Spurk, D., & Volmer, J. (2011). The construct of career success: Measurement issues and an empirical example. Journal for Labour Market Research, 43(3), 195–206. DOI: 10.1007/s12651-010-0034-6

[2] Aßmann, C., Steinhauer, H. W., Würbach, A., Zinn, S., Hammon, A., Kiesl, H., Rohwer, G., Rässler, S., & Blossfeld, H.-P. (2019). Sampling designs of the National Educational Panel Study: Setup and panel development. In H.-P. Blossfeld & H.-G. Roßbach (Eds.), Education as a lifelong process: The German National Educational Panel Study (NEPS) (2nd ed., pp. 35–55). Springer VS. DOI: 10.1007/978-3-658-23162-0_3

[3] Allmendinger, J., Kleinert, C., Pollak, R., Vicari, B., Wölfel, O., Althaber, A., Antoni, M., Christoph, B., Drasch, K., Janik, F., Künster, R., Laible, M.-C., Leuze, K., Matthes, B., Ruland, M., Schulz, B., & Trahms, A. (2019). Adult education and lifelong learning. In H.-P. Blossfeld & H.-G. Roßbach (Eds.), Education as a lifelong process: The German National Educational Panel Study (NEPS) (2nd ed., pp. 325–346). Springer VS. DOI: 10.1007/978-3-658-23162-0_17

[4] Arens, A. K., & Morin, A. J. S. (2016). Relations between teachers’ emotional exhaustion and students’ educational outcomes. Journal of Educational Psychology, 108(6), 800–813. DOI: 10.1037/edu0000105

[5] Bachbauer, N., Wolf, C., & Fuß, D. (2022). Education and employment trajectories in NEPS-ADIAB: The survey data of the National Educational Panel Study linked to administrative data of the Institute for Employment Research. European Sociological Review, 38(4), 663–676. DOI: 10.1093/esr/jcab057

[6] Bäumer, T., Klieme, E., Kuger, S., Maaz, K., Roßbach, H.-G., Stecher, L., & Struck, O. (2019). Education processes in life-course-specific learning environments. In H.-P. Blossfeld & H.-G. Roßbach (Eds.), Education as a lifelong process: The German National Educational Panel Study (NEPS) (2nd ed., pp. 83–99). Springer VS. DOI: 10.1007/978-3-658-23162-0_5

[7] Baumert, J., Blum, W., Brunner, M., Dubberke, T., Jordan, A., Klusmann, U., Krauss, S., Kunter, M., Löwen, K., Neubrand, M., & Tsai, Y.-M. (2008). Professionswissen von Lehrkräften, kognitiv aktivierender Mathematikunterricht und die Entwicklung von mathematischer Kompetenz (COACTIV): Dokumentation der Erhebungsinstrumente [Professional competence of teachers, cognitively activating instruction, and the development of students’ mathematical literacy (COACTIV): Documentation of the survey instruments]. Materialien aus der Bildungsforschung: Vol. 83. Max-Planck-Institut für Bildungsforschung. https://pure.mpg.de/pubman/item/item_2100057_8/component/file_2197666/Materialien_Bildungsforschung_MPIB_083.pdf

[8] Baumert, J., & Kunter, M. (2013). The COACTIV model of teachers’ professional competence. In M. Kunter, J. Baumert, W. Blum, U. Klusmann, S. Krauss, & M. Neubrand (Eds.), Cognitive activation in the mathematics classroom and professional competence of teachers: Results from the COACTIV project (pp. 25–48). Springer. DOI: 10.1007/978-1-4614-5149-5_2

[9] Beaton, A. E., & Barone, J. L. (2017). Large-scale group-score assessment. In R. E. Bennett & M. von Davier (Eds.), Advancing human assessment: The methodological, psychological and policy contributions of ETS (pp. 233–284). Springer. DOI: 10.1007/978-3-319-58689-2_8

[10] Beaton, A. E., & Zwick, R. (1990). The effect of changes in the national assessment: Disentangling the NAEP 1985–86 reading anomaly. Revised. (NAEP Report No. 17-TR-21). Princeton, NJ: Educational Testing Service. http://files.eric.ed.gov/fulltext/ED322216.pdf

[11] Bela, D. (2016). Applied large-scale data editing. In H.-P. Blossfeld, J. von Maurice, M. Bayer, & J. Skopek (Eds.), Methodological issues of longitudinal surveys: The example of the National Educational Panel Study (pp. 649–667). Springer VS. DOI: 10.1007/978-3-658-11994-2_36

[12] Besa, K.-S., & Vietgen, S. (2017). Repräsentanz, Integration und Abbruchintentionen von Studierenden mit Migrationshintergrund in Lehramtsstudiengängen: Eine Analyse anhand der Daten des Nationalen Bildungspanels (NEPS) [Representativeness, integration and dropout intentions of immigrant students in teacher preparation programmes: An analysis of NEPS data]. Beiträge zur Lehrerinnen- und Lehrerbildung, 35(1), 195–205. DOI: 10.36950/bzl.35.2017.9482

[13] Blömeke, S., Houang, T., Hsieh, F.-J., & Wang, T.-Y. (2017). Effects of job motives, teacher knowledge, and school context on beginning teachers’ commitment to stay in the profession. G. K. LeTendre & M. Akiba (Eds.), International handbook of teacher quality and policy (pp. 374–387). Routledge. DOI: 10.4324/9781315710068-24

[14] Blömeke, S., & Klein, P. (2013). When is a school environment perceived as supportive by beginning mathematics teachers? Effects of leadership, trust, autonomy and appraisal on teaching quality. International Journal of Science and Mathematics Education, 11, 1029–1048. DOI: 10.1007/s10763-013-9424-x

[15] Blossfeld, H.-P., & Roßbach, H.-G. (Eds.). (2019). Education as a lifelong process: The German National Educational Panel Study (NEPS) (2nd ed.). Springer VS. DOI: 10.1007/978-3-658-23162-0

[16] Blossfeld, H.-P., Schneider, T., & Doll, J. (2009). Methodological advantages of panel studies. Journal for Educational Research Online, 1(1), 10–32. https://www.waxmann.com/artikelART102638

[17] Blossfeld, H.-P., von Maurice, J., Bayer, M., & Skopek, J. (Eds.). (2016). Methodological issues of longitudinal surveys: The example of the National Educational Panel Study. Springer VS. DOI: 10.1007/978-3-658-11994-2

[18] Bos, W., Lankes, E.-M., Prenzel, M., Schwippert, K., Valtin, R., Voss, A., & Walther, G. (Eds.). (2005). IGLU: Skalenhandbuch zur Dokumentation der Erhebungsinstrumente [IGLU: Scale manual for the documentation of the survey instruments]. Waxmann.

[19] Bos, W., Strietholt, R., Goy, M., Stubbe, T. C., Tarelli, I., & Hornberg, S. (2010). IGLU 2006. Dokumentation der Erhebungsinstrumente [IGLU 2006. Documentation of survey instruments]. Waxmann.

[20] Brachem, J.-C., Aschinger, F., Fehring, G., Grotheer, M., Herrmann, S., Kühn, M., Liebeskind, U., Ortenburger, A., & Schaeper, H. (2019). Higher education and the transition to work. In H.-P. Blossfeld & H.-G. Roßbach (Eds.), Education as a lifelong process: The German National Educational Panel Study (NEPS) (2nd ed., pp. 297–323). Springer VS. DOI: 10.1007/978-3-658-23162-0_16

[21] Bush, T., & Glover, D. (2014). School leadership models: What do we know? School Leadership & Management, 34(5), 553–571. DOI: 10.1080/13632434.2014.928680

[22] Carstensen, B., & Klusmann, U. (2021). Assertiveness and adaptation: Prospective teachers’ social competence development and its significance for occupational well-being. The British Journal of Educational Psychology, 91(1), 500–526. DOI: 10.1111/bjep.12377 32914428

[23] Cookson, P. S. (1986). A framework for theory and research on adult education participation. Adult Education Quarterly, 36(3), 130–141. DOI: 10.1177/0001848186036003002

[24] Dizinger, V., & Böhm-Kasper, O. (2019). Interprofessionelle Kooperation [Interprofessional cooperation]. M. Harring, C. Rohlfs, & M. Gläser-Zikuda (Eds.), Handbuch Schulpädagogik (pp. 752–764). Waxmann.

[25] Drüge, M., Schleider, K., & Rosati, A.-S. (2014). Psychosoziale Belastungen im Referendariat – Merkmale, Ausprägungen, Folgen [Psychosocial hazards of trainee teachers – characteristics, manifestations, outcomes]. DDS – Die Deutsche Schule, 106(4), 358–372.

[26] Enzmann, D., & Kleiber, D. (1989). Helfer-Leiden: Stress und Burnout in psychosozialen Berufen [Helpers’ ordeals: Stress and burnout in the human services professions]. Asanger.

[27] Europäische Kommission/EACEA/Eurydice. (2015). Der Lehrerberuf in Europa: Praxis, Wahrnehmungen und politische Maßnahmen [The teaching profession in Europe: Practices, perceptions, and policies]. Eurydice-Bericht. Amt für Veröffentlichungen der Europäischen Union. DOI: 10.2797/166767

[28] Ewen, C. P. J. (2013). Politische Fertigkeiten im Führungskontext. [Political skill in leadership context] [Dissertation]. Universität Bonn, Bonn. http://hss.ulb.uni-bonn.de/2013/3115/3115.pdf

[29] Fend, H. (2006). Neue Theorie der Schule. Einführung in das Verstehen von Bildungssystemen [New theory of school. Introduction to the understanding of education systems]. VS Verlag für Sozialwissenschaften.

[30] Fickermann, D., & Edelstein, B. (Eds.) (2021). Schule während der Corona-Pandemie. Neue Ergebnisse und Überblick über ein dynamisches Forschungsfeld [Schooling during the Corona pandemic. New results and an overview of a dynamic field of research]. *DDS Die Deutsche Schule Beiheft: Vol. 17*. Waxmann. DOI: 10.31244/9783830993315

[31] Fuß, D., & Wenzig, K. (2019). The research data center: Making National Educational Panel Study data available for research. In H.-P. Blossfeld & H.-G. Roßbach (Eds.), Education as a lifelong process: The German National Educational Panel Study (NEPS) (2nd ed., 361–378). Springer VS. DOI: 10.1007/978-3-658-23162-0_19

[32] Fussangel, K., Rürup, M., & Gräsel, C. (2016). Lehrerfortbildung als Unterstützungssystem [Teacher professional development as a support system]. In H. Altrichter & K. Maag Merki (Eds.), Handbuch Neue Steuerung im Schulsystem (2nd ed., pp. 361–384). Springer. DOI: 10.1007/978-3-531-18942-0_13

[33] Gagarina, L., & Saldern, M. von. (2010). Professionalisierung der Lehrkräfte [Professionalisation of teachers]. In M. Demmer & M. von Saldern (Eds.), “Helden des Alltags”. Erste Ergebnisse der Schulleitungs- und Lehrkräftebefragung (TALIS) in Deutschland (pp. 47–63). Waxmann.

[34] Gebhardt, M., Schwab, S., Krammer, M., Gasteiger-Klicpera, B., & Sälzer, C. (2014). Erfassung von individualisiertem Unterricht in der Sekundarstufe I: Eine Quantitative Überprüfung der Skala “Individualisierter Unterricht” in zwei Schuluntersuchungen in der Steiermark [Measuring teaching practices in inclusive settings in secondary classrooms: A quantitative verification of the scale “individualized teaching” in two school surveys in Styria]. Zeitschrift für Bildungsforschung, 4(3), 303–316. DOI: 10.1007/s35834-014-0095-7

[35] Gräsel, C., Fußangel, K., & Pröbstel, C. (2006). Lehrkräfte zur Kooperation anregen – eine Aufgabe für Sisyphos? [Prompting teachers to cooperate – a Sisyphean task?] Zeitschrift für Pädagogik, 52(2), 205–219. DOI: 10.25656/01:4453

[36] Greubel, M. (2017). Die Einführung einer erweiterten Schulleitung an eigenverantwortlichen beruflichen Schulen [Introduction of a distributed leadership at autonomous vocational schools] [Dissertation]. Friedrich-Alexander-Universität Erlangen-Nürnberg, Nürnberg, Germany. DOI: 10.25593/978-3-7450-9071-0

[37] Gross, C., Bela, A., Jungbauer-Gans, M., Jobst, A., & Schwarze, J. (2019). Educational returns over the life course. In H.-P. Blossfeld & H.-G. Roßbach (Eds.), Education as a lifelong process: The German National Educational Panel Study (NEPS) (2nd ed., pp. 137–153). Springer VS. DOI: 10.1007/978-3-658-23162-0_8

[38] Gülen, Ş. (2021). Lehramtsstudium mit Migrationshintergrund: Einflussfaktoren auf die Studienfachentscheidung und den Studienverlauf [Teacher training with migrant background: Factors influencing the choice and course of studies]. Springer. DOI: 10.1007/978-3-658-32882-5

[39] Gülen, Ş. (2022). Ursachen der Unterrepräsentanz von Lehrkräften mit Migrationshintergrund an Schulen in Deutschland. Eine theoretische und empirische Analyse des Studienwahlverhaltens [Causes of the underrepresentation of teachers with a migrant background at schools in Germany. A theoretical and empirical analysis of study choice behaviour]. In M. Stock, N. Hodaie, S. Immerfall, & M. Menz (Eds.), Migration – Gesellschaft – Schule. Arbeitstitel: Migrationsgesellschaft: Pädagogik – Profession – Praktik (pp. 105–125). Springer VS. DOI: 10.1007/978-3-658-34087-2_6

[40] Hagmaier, T., Abele, A. E., & Goebel, K. (2018). How do career satisfaction and life satisfaction associate? Journal of Managerial Psychology, 33(2), 142–160. DOI: 10.1108/JMP-09-2017-0326

[41] Hartmann, F. G., & Ertl, B. (2021). Big Five personality trait differences between students from different majors aspiring to the teaching profession. Current Psychology. Advance online publication. DOI: 10.1007/s12144-021-02528-3

[42] Hartmann, F. G., Mouton, D., & Ertl, B. (2022). The Big Six interests of STEM and non-STEM students inside and outside of teacher education. Teaching and Teacher Education, 112, Article 103622. DOI: 10.1016/j.tate.2021.103622

[43] Hattie, J. (2009). Visible learning: A synthesis of over 800 meta-analyses relating to achievement. Routledge.

[44] Heinitz, K., & Rowold, J. (2007). Gütekriterien einer deutschen Adaptation des Transformational Leadership Inventory (TLI) von Podsakoff [Psychometric properties of a German adaptation of the Transformational Leadership Inventory (TLI) by Podsakoff]. Zeitschrift für Arbeits- und Organisationspsychologie, 51(1), 1–15. DOI: 10.1026/0932-4089.51.1.1

[45] Helgeson, V. S. (2003). Social support and quality of life. Quality of Life Research, 12(Suppl. 1), 25–31. DOI: 10.1023/A:1023509117524 12803308

[46] Helmke, A. (2012). Unterrichtsqualität und Lehrerprofessionalität: Diagnose, Evaluation und Verbesserung des Unterrichts [Teaching quality and teachers’ professionalism: Diagnosis, evaluation and improvement of teaching] (4th ed.). Kallmayer.

[47] Hilton, J. L., & von Hippel, W. (1996). Stereotypes. Annual Review of Psychology, 47(1), 237–271. DOI: 10.1146/annurev.psych.47.1.237 15012482

[48] Hußmann, A., Wendt, H., Bos, W., Bremerich-Vos, A., Kasper, D., Lankes, E.-M., McElvany, N., Stubbe, T. C., & Valtin, R. (Eds.) (2017). IGLU 2016: Lesekompetenzen von Grundschulkindern in Deutschland im internationalen Vergleich [IGLU 2016: Reading competencies of primary school children in Germany in international comparison]. Waxmann.

[49] Hüttges, A., & Fay, D. (2019). Proaktives Verhalten: Schlüsselkompetenz für die Karriereentwicklung [Proactive behaviour: Key competence for career development]. S. Kauffeld & D. Spurk (Eds.), Handbuch Karriere und Laufbahnmanagement (pp. 487–509). Springer. DOI: 10.1007/978-3-662-48750-1_20

[50] Judge, T. A., & Piccolo, R. F. (2004). Transformational and transactional leadership: A meta-analytic test of their relative validity. Journal of Applied Psychology, 89(5), 755–768. DOI: 10.1037/0021-9010.89.5.755 15506858

[51] Klassen, R. M., & Chiu, M. M. (2011). The occupational commitment and intention to quit of practicing and pre-service teachers: Influence of self-efficacy, job stress, and teaching context. Contemporary Educational Psychology, 36(2), 114–129. DOI: 10.1016/j.cedpsych.2011.01.002

[52] Klieme, E., Lipowsky, F., Rakoczy, K., & Ratzka, N. (2006). Qualitätsdimensionen und Wirksamkeit von Mathematikunterricht. Theoretische Grundlagen und ausgewählte Ergebnisse des Projekts “Pythagaros” [Quality dimensions and effectiveness of mathematics instruction. Theoretical foundations and selected findings of the “Pythagoras” project]. M. Prenzel & L. Allolio-Näcke (Eds.), Untersuchungen zur Bildungsqualität von Schule. Abschlussbericht des DFG-Schwerpunktprogramms (pp. 127–146). Waxmann.

[53] Klusmann, U., Kunter, M., Trautwein, U., Lüdtke, O., & Baumert, J. (2008). Teachers’ occupational well-being and quality of instruction: The important role of self-regulatory patterns. Journal of Educational Psychology, 100(3), 702–715. DOI: 10.1037/0022-0663.100.3.702

[54] Klusmann, U., Kunter, M., Voss, T., & Baumert, J. (2012). Berufliche Beanspruchung angehender Lehrkräfte: Die Effekte von Persönlichkeit, pädagogischer Vorerfahrung und professioneller Kompetenz [Emotional exhaustion and job satisfaction of beginning teachers: The role of personality, educational experience and professional competence]. Zeitschrift für Pädagogische Psychologie, 26(4), 275–290. DOI: 10.1024/1010-0652/a000078

[55] Klusmann, U., & Richter, D. (2014). Beanspruchungserleben von Lehrkräften und Schülerleistung. Eine Analyse des IQB-Ländervergleichs in der Primarstufe [Teachers’ experience of stress and student performance – An analysis of the comparison between the German Laender on the level of primary education carried out by the IQB (Institute for Educational Quality Improvement)]. Zeitschrift für Pädagogik, 60(2), 202–224.

[56] Klusmann, U., Richter, D., & Lüdtke, O. (2016). Teachers’ emotional exhaustion is negatively related to students’ achievement: Evidence from a large-scale assessment study. Journal of Educational Psychology, 108(8), 1193–1203. DOI: 10.1037/edu0000125

[57] Kultusministerkonferenz. (2014). Standards für die Lehrerbildung: Bildungswissenschaften [Standards for teacher education: Educational sciences] [Beschluss der Kultusministerkonferenz vom 16.12.2004 i. d. F. vom 12.06.2014]. http://www.kmk.org/fileadmin/veroeffentlichungen_beschluesse/2004/2004_12_16-Standards-Lehrerbildung-Bildungswissenschaften.pdf

[58] Kunter, M., Baumert, J., Leutner, D., Terhart, E., Seidel, T., Dicke, T., Kunina-Habenicht, O., Linninger, C., Lohse-Bossenz, H., Schulze-Stocker, F., & Stürmer, K. (2017). Dokumentation der Erhebungsinstrumente der Projektphasen des BilWiss-Forschungsprogramms von 2009 bis 2016 [Documentation of the survey instruments of the BilWIss research programme 2009 to 2016]. Frankfurt a. M. IQB. https://www.iqb.hu-berlin.de/fdz/studies/BilWiss/BilWiss_Skalenha.pdf

[59] Kunter, M., Frenzel, A., Nagy, G., Baumert, J., & Pekrun, R. (2011). Teacher enthusiasm: Dimensionality and context specificity. Contemporary Educational Psychology, 36(4), 289–301. DOI: 10.1016/j.cedpsych.2011.07.001

[60] Kunter, M., Klusmann, U., Baumert, J., Richter, D., Voss, T., & Hachfeld, A. (2013). Professional competence of teachers: Effects on instructional quality and student development. Journal of Educational Psychology, 105(3), 805–820. DOI: 10.1037/a0032583

[61] Kunter, M., & Pohlmann, B. (2015). Lehrer [Teachers]. In E. Wild & J. Möller (Eds.), Pädagogische Psychologie (2nd ed., pp. 261–281). Springer. DOI: 10.1007/978-3-642-41291-2_11

[62] Laschke, C., & König, J. (2014). Erfassung der Lerngelegenheiten (opportunities to learn, OTL) [Measuring learning opportunities]. In C. Laschke & S. Blömeke (Eds.), Teacher Education and Development Study: Learning to teach mathematics (TEDS-M 2008). Dokumentation der Erhebungsinstrumente (S. 45–108). Waxmann.

[63] Lauterbach, O. (2016). Erfassung wirtschaftswissenschaftlicher Fachkompetenz von Studierenden in Startkohorte 5 des Nationalen Bildungspanels – Technischer Bericht (Aktualisierung) [Assessment of students’ professional competence in economics in Starting Cohort 5 of the National Educational Panel: Technical Report] (NEPS Working Paper No. 51). Bamberg, Germany: Leibniz-Institut für Bildungsverläufe. https://www.neps-data.de/Portals/0/Working%20Papers/Aktualisierung_WP_LI_20160901.pdf

[64] Leithwood, K., & Sun, J. (2012). The nature and effects of transformational school leadership. Educational Administration Quarterly, 48(3), 387–423. DOI: 10.1177/0013161X11436268

[65] Liebeskind, U., & Vietgen, S. (2017). Panelausfall in der Studierendenkohorte des Nationalen Bildungspanels: Analyse des Ausfallprozesses zwischen der ersten und zweiten telefonischen Befragung [Panel dropout in the student cohort of the National Educational Panel: Analysis of the dropout process between the first and second telephone survey] (NEPS Working Paper No. 70). Bamberg, Germany: Leibniz-Institut für Bildungsverläufe, Nationales Bildungspanel. https://www.neps-data.de/Portals/0/WorkingPapers/WP_LXX.pdf

[66] LIfBi. (2016). Starting Cohort 4: Grade 9 (SC4). Waves 3 and 4. Questionnaires (SUF Version 4.0.0). Bamberg, Germany: Leibniz Institute for Educational Trajectories (LIfBi). https://www.neps-data.de/Portals/0/NEPS/Datenzentrum/Forschungsdaten/SC4/4-0-0/SC4_4-0-0_en.pdf

[67] Lipowsky, F., & Rzejak, D. (2019). Was macht Fortbildungen für Lehrkräfte erfolgreich? – Ein Update [What makes further training for teachers successful? An update]. B. Groot-Wilken & R. Koerber (Eds.), Nachhaltige Professionalisierung für Lehrerinnen und Lehrer: Ideen, Entwicklungen, Konzepte (pp. 15–56). wbv Media.

[68] Little, J. W. (1990). The persistence of privacy: Autonomy and initiative in teachers’ professional relations. Teachers College Record, 91(4), 509–536. DOI: 10.1177/016146819009100403

[69] Lorenz, R., Lepper, C., Brüggemann, T., & McElvany, N. (2020). Unterricht während der Corona-Pandemie. Lehrkräftebefragung. Ergebnisse, Teil I “Der Unterricht” [Teaching during the Corona pandemic. Survey of teachers. Results, part I “Instruction”]. Dortmund, Germany. Institut für Schulentwicklungsforschung (IFS), Universität Dortmund. https://www.tu-dortmund.de/storages/zentraler_bilderpool/user_upload/UCP_Kurzbericht_final.pdf

[70] Lucksnat, C., Richter, E., Klusmann, U., Kunter, M., & Richter, D. (2020). Unterschiedliche Wege ins Lehramt – unterschiedliche Kompetenzen? Ein Vergleich von Quereinsteigern und traditionell ausgebildeten Lehramtsanwärtern im Vorbereitungsdienst [Different ways into teaching – different competences? A comparison of alternatively certified teachers and traditionally certified teachers in the induction phase]. Zeitschrift für Pädagogische Psychologie (advance online publication), 1–16. DOI: 10.1024/1010-0652/a000280

[71] Maslach, C., & Jackson, S. (1981). The measurement of experienced burnout. Journal of Occupational Behavior, 2(2), 99–113. DOI: 10.1002/job.4030020205

[72] Maslach, C., Schaufeli, W. B., & Leiter, M. P. (2001). Job burnout. Annual Review of Psychology, 52, 397–422. DOI: 10.1146/annurev.psych.52.1.397 11148311

[73] Menge, C., Euler, T., & Schaeper, H. (2021). Überzeugungen und Selbstwirksamkeitserwartungen zum inklusiven Unterricht bei (angehenden) Lehrkräften: der Einfluss von Lerngelegenheiten [Beliefs and perceived self-efficacy of (prospective) teachers regarding inclusive education: The effect of learning opportunities]. Zeitschrift für Erziehungswissenschaft, 24(6), 1283–1308. DOI: 10.1007/s11618-021-01038-z

[74] Menge, C., & Schaeper, H. (2019). Berufliche Selbstregulation von Lehrkräften: Überprüfung eines Kurzinstruments [Teachers’ occupational self-regulation: Validation of a short measurement instrument]. Zeitschrift für Erziehungswissenschaft, 22(6), 1489–1513. DOI: 10.1007/s11618-018-0851-x

[75] NEPS Network. (2022). National Educational Panel Study, Scientific Use File of Starting Cohort First-Year Students. Bamberg: Leibniz Institute for Educational Trajectories (LIfBi). DOI: 10.5157/NEPS:SC5:17.0.0

[76] Neugebauer, M. (2020). Leistungsmerkmale [Performance characteristics]. C. Cramer, J. König, M. Rothland, & S. Blömeke (Eds.), Handbuch Lehrerinnen- und Lehrerbildung (pp. 798–803). Julius Klinkhardt. DOI: 10.35468/hblb2020-098

[77] OECD. (Ed.). (2009). Creating effective teaching and learning environments: First results from TALIS. OECD.

[78] Ohly, S., Sonnentag, S., & Pluntke, F. (2006). Routinization, work characteristics and their relationships with creative and proactive behaviors. Journal of Organizational Behavior, 27(3), 257–279. DOI: 10.1002/job.376

[79] Olczyk, M., Will, G., & Kristen, C. (2016). Immigrants in the NEPS: Identifying generation status and group of origin (2nd ed.) (NEPS Survey Paper No. 4). Bamberg, Germany: Leibniz Institute for Educational Trajectories, National Educational Panel Study. DOI: 10.5157/NEPS:SP04:1.0

[80] Osada, J.-C., & Schaeper, H. (2021). Individual characteristics of teacher education students: Re-examining the negative selection hypothesis. Journal for Educational Research Online, 13(2), 109–131. DOI: 10.31244/jero.2021.02.06

[81] Pajares, M. F. (1992). Teachers’ beliefs and educational research: Cleaning up a messy construct. Review of Educational Research, 62(3), 307–332. DOI: 10.3102/00346543062003307

[82] Pietsch, M., Scholand, B., Graw, S., Hengstmann, E., & Kulin, S. (2014). Skalenhandbuch der Schulinspektion Hamburg: Fragebögen für Pädagoginnen und Pädagogen, Eltern und Schülerinnen und Schüler (gültig ab 15.11.2013) [Scale handbook of the Hamburg school inspection: Questionnaires for teachers, parents and students (valid from 15.11.2013)]. Hamburg, Germany: Institut für Bildungsmonitoring und Qualitätsentwicklung (ifbq).

[83] Pietsch, M., & Tulowitzki, P. (2017). Disentangling school leadership and its ties to instructional practices – an empirical comparison of various leadership styles. School Effectiveness and School Improvement, 28(4), 629–649. DOI: 10.1080/09243453.2017.1363787

[84] Podsakoff, P. M., Mackenzie, S. B., Moorman, R. H., & Fetter, R. (1990). Transformational leader behaviors and their effects on followers’ trust in leader, satisfaction, and organizational citizenship behaviors. The Leadership Quarterly, 1(2), 107–142. DOI: 10.1016/1048-9843(90)90009-7

[85] Pohlmann, B., & Möller, J. (2010). Fragebogen zur Erfassung der Motivation für die Wahl des Lehramtsstudiums (FEMOLA) [Motivation for choosing teacher education questionnaire]. Zeitschrift für Pädagogische Psychologie, 24(1), 73–84. DOI: 10.1024/1010-0652/a000005

[86] Radisch, F., Stecher, L., Klieme, E., & Kühnbach, O. (2007). Unterrichts- und Angebotsqualität aus Schülersicht [Quality of teaching and quality of extracurricular activities from the students’ perspective]. H.-G. Holtappels, E. Klieme, T. Rauschenbach, & L. Stecher (Eds.), Ganztagsschule in Deutschland. Ergebnisse der Ausgangserhebung der “Studie zur Entwicklung von Ganztagsschulen” (StEG) (pp. 227–260). Juventa.

[87] Retelsdorf, J., Bauer, J., Gebauer, S. K., Kauper, T., & Möller, J. (2014). Erfassung berufsbezogener Selbstkonzepte von angehenden Lehrkräften (ERBSE-L) [Measuring prospective teachers’ professional self-concept]. Diagnostica, 60(2), 98–110. DOI: 10.1026/0012-1924/a000108

[88] Retelsdorf, J., & Möller, J. (2012). Grundschule oder Gymnasium? Zur Motivation ein Lehramt zu studieren [Primary or secondary school? On the motivation for choosing teacher education]. Zeitschrift für Pädagogische Psychologie, 26(1), 5–17. DOI: 10.1024/1010-0652/a000056

[89] Richter, D., Böhme, K., Bastian-Wurzel, J., Anand Pant, H., & Stanat, P. (2014). IQB-Ländervergleich 2011: Skalenhandbuch zur Dokumentation der Erhebungsinstrumente [IQB National Assessment Study 2011 scaling manual. Documentation of the survey instruments]. Humboldt-Universität zu Berlin, Institut zur Qualitätsentwicklung im Bildungswesen. DOI: 10.18452/3127

[90] Richter, D., Engelbert, M., Weirich, S., & Anand Pant, H. (2013). Differentielle Teilnahme an Lehrerfortbildungen und deren Zusammenhang mit professionsbezogenen Merkmalen von Lehrkräften. [Differential use of professional development programs and its relationship to professional characteristics of teachers]. Zeitschrift für Pädagogische Psychologie, 27(3), 193–207. DOI: 10.1024/1010-0652/a000104

[91] Richter, D., Kunter, M., Anders, Y., Klusmann, U., Lüdtke, O., & Baumert, J. (2010). Inhalte und Prädiktoren beruflicher Fortbildung von Mathematiklehrkräften [Content and predictors of professional development activities of mathematics teachers]. Empirische Pädagogik, 24(2), 151–168.

[92] Richter, D., Kunter, M., Lüdtke, O., Klusmann, U., & Baumert, J. (2011). Soziale Unterstützung beim Berufseinstieg ins Lehramt: Eine empirische Untersuchung zur Bedeutung von Mentoren und Mitreferendaren [Social support at career entry for teachers—An empirical study on the importance of mentors and peers]. Zeitschrift für Erziehungswissenschaft, 14(1), 35–59. DOI: 10.1007/s11618-011-0173-8

[93] Robinson, V. M. J., Lloyd, C. A., & Rowe, K. J. (2008). The impact of leadership on student outcomes: An analysis of the differential effects of leadership types. Educational Administration Quarterly, 44(5), 635–674. DOI: 10.1177/0013161X08321509

[94] Rochnia, M., Trempler, K., & Schellenbach-Zell, J. (2019). Vergleich der Forschungs- sowie Praxisorientierung zwischen Lehramts- und Medizinstudium [Comparison of research and practice orientation between teacher and medical training]. Zeitschrift für empirische Hochschulforschung, 2(2), 123–138. DOI: 10.3224/zehf.v3i2.03

[95] Schaarschmidt, U., & Fischer, A. W. (2001). Bewältigungsmuster im Beruf [Coping patterns at work]. Vandenhoeck & Ruprecht.

[96] Schaeper, H., & Weiß, T. (2016). The conceptualization, development, and validation of an instrument for measuring the formal learning environment in higher education. In H.-P. Blossfeld, J. von Maurice, M. Bayer, & J. Skopek (Eds.), Methodological issues of longitudinal surveys: The example of the National Educational Panel Study (pp. 269–292). Springer VS. DOI: 10.1007/978-3-658-11994-2_16

[97] Schier, A., Bender, M., Koberg, T., Bogensperger, B., Gruner, S., Schiller, D., von Maurice, J., & Engelhardt-Wölfler, H. (2019). Data protection issues in the National Educational Panel Study. In H.-P. Blossfeld & H.-G. Roßbach (Eds.), Education as a lifelong process: The German National Educational Panel Study (NEPS) (2nd ed., 347–359). Springer VS. DOI: 10.1007/978-3-658-23162-0_18

[98] Schönberger, K., & Koberg, T. (2017). Regional data: Microm. (NEPS Research Data). Bamberg, Germany: Leibniz Institute for Educational Trajectories (LIfBi). https://www.neps-data.de/Portals/0/NEPS/Datenzentrum/Forschungsdaten/Regio_Microm_en.pdf

[99] Schotte, K., Rjosk, C., Edele, A., Hachfeld, A., & Stanat, P. (2022). Do teachers’ cultural beliefs matter for students’ school adaptation? A multilevel analysis of students’ academic achievement and psychological school adjustment. Social Psychology of Education, 25(1), 75–112. DOI: 10.1007/s11218-021-09669-0

[100] Senkbeil, M., Ihme, J. M., & Schöber, C. (2021). Schulische Medienkompetenzförderung in einer digitalen Welt: Über welche digitalen Kompetenzen verfügen angehende Lehrkräfte? [Dissemination of media literacy at school in a digital world: Are teacher candidates digitally competent?]. Psychologie in Erziehung Und Unterricht, 68(1), 4–22. DOI: 10.2378/peu2020.art12d

[101] Skaalvik, E. M., & Skaalvik, S. (2011). Teacher job satisfaction and motivation to leave the teaching profession: Relations with school context, feeling of belonging, and emotional exhaustion. Teaching and Teacher Education, 27(6), 1029–1038. DOI: 10.1016/j.tate.2011.04.001

[102] Skaalvik, E. M., & Skaalvik, S. (2017). Motivated for teaching? Associations with school goal structure, teacher self-efficacy, job satisfaction and emotional exhaustion. Teaching and Teacher Education, 67, 152–160. DOI: 10.1016/j.tate.2017.06.006

[103] Skopek, J., Wenzig, K., Bela, D., Koberg, T., Munz, M., & Fuß, D. (2016). Data dissemination, documentation, and user support. In H.-P. Blossfeld, J. von Maurice, M. Bayer, & J. Skopek (Eds.), Methodological issues of longitudinal surveys: The example of the National Educational Panel Study (pp. 597–609). Springer VS. DOI: 10.1007/978-3-658-11994-2_33

[104] Stiftung Universität Hildesheim. (2016). Fragebogen für Lehrerinnen und Lehrer an Grund- und Förderschulen des Projekts “Einstellungen zur Inklusion in der Schule” (Projekt E1NS). Unpublished [Questionnaire for teachers at primary and special education schools of the project “Attitudes towards inclusive education in schools”]. Hildesheim: Universität Hildesheim, Institut für Psychologie/Institut für Grundschuldidaktik und Sachunterricht.

[105] Sturm, M., Reiher, S., Heinitz, K., & Soellner, R. (2011). Transformationale, transaktionale und passiv-vermeidende Führung: Eine metaanalytische Untersuchung ihres Zusammenhangs mit Führungserfolg [Transformational and transactional leadership – A meta-analytical examination of their relationship with leadership effectiveness]. Zeitschrift für Arbeits- und Organisationspsychologie, 55(2), 88–104. DOI: 10.1026/0932-4089/a000049

[106] Thomas, J. P., Whitman, D. S., & Viswesvaran, C. (2010). Employee proactivity in organizations: A comparative meta-analysis of emergent proactive constructs. Journal of Occupational and Organizational Psychology, 83(2), 275–300. DOI: 10.1348/096317910X502359

[107] van Dyne, L., & LePine, J. A. (1998). Helping and voice extra-role behaviors: Evidence of construct and predictive validity. Academy of Management Journal, 41(1), 108–119. DOI: 10.2307/256902

[108] Vennemann, M., Eickelmann, B., Labusch, A., & Drossel, K. (2021). ICILS 2018 #Deutschland: Dokumentation der Erhebungsinstrumente der zweiten Computer and Information Literacy Study [ICILS 2018 #Germany: Documentation of the survey instruments used in the second Computer and Information Literacy Study]. Waxmann. DOI: 10.31244/9783830993278

[109] von Maurice, J., Blossfeld, H.-P., Roßbach, H.-G. (2016). The National Educational Panel Study: Milestones of the years 2006 to 2015. In: H.-P. Blossfeld, J. von Maurice, M. Bayer, & J. Skopek. (Eds.), Methodological issues of longitudinal surveys: The example of the National Educational Panel Study (pp. 3–18). Springer VS. DOI: 10.1007/978-3-658-11994-2_1

[110] Watt, H. M. G., Richardson, P. W., Klusmann, U., Kunter, M., Beyer, B., Trautwein, U., & Baumert, J. (2012). Motivations für choosing teaching as a career. An international comparison using the FIT-Choice scale. Teaching and Teacher Education, 28(6), 791–805. DOI: 10.1016/j.tate.2012.03.003

[111] Weber, A. (2014). Data manual Starting Cohort 5: Context data. Hannover, Germany: DZHW. https://www.neps-data.de/Portals/0/NEPS/Datenzentrum/Forschungsdaten/SC5/4-0-0/SC5_ContextData.pdf

[112] Weinert, S., Artelt, C., Prenzel, M., Senkbeil, M., Ehmke, T., Carstensen, C. H., & Lockl, K. (2019). Development of competencies across the life course. In H.-P. Blossfeld & H.-G. Roßbach (Eds.), Education as a lifelong process: The German National Educational Panel Study (NEPS) (2nd ed., pp. 57–81). Springer VS. DOI: 10.1007/978-3-658-23162-0_4

[113] Weiss, H. M. (2002). Deconstructing job satisfaction. Human Resource Management Review, 12(2), 173–194. DOI: 10.1016/S1053-4822(02)00045-1

[114] Wenz, S. E. (2020). Discrimination in education [Dissertation]. Bamberg, Germany: Otto-Friedrich-Universität Bamberg. DOI: 10.21241/SSOAR.67307

[115] Wenz, S. E., Olczyk, M., & Lorenz, G. (2016). Measuring teachers’ stereotypes in the NEPS (NEPS Survey Paper No. 3). Bamberg, Germany: Leibniz Institute for Educational Trajectories, National Educational Panel Study. DOI: 10.2139/ssrn.2999297

[116] Weresch-Deperrois, I., Bodensohn, R., & Jäger, R. S. (2009). KOSTA – Ein Instrument zur Kompetenz- und Standardorientierung in der Lehrerbildung: Skalenhandbuch [KOSTA – A competence and standard based instrument in teacher training: Scale manual]. Landau, Germany: Universität Koblenz-Landau. http://www.uni-landau.de/schulprakt-studien/091215_Skalenhandbuch_KOSTA1.pdf

[117] Windlinger, R., Warwas, J., & Hostettler, U. (2020). Dual effects of transformational leadership on teacher efficacy in close and distant leadership situations. School Leadership & Management, 40(1), 64–87. DOI: 10.1080/13632434.2019.1585339

[118] Wohlkinger, F., Blumenfelder, A. R., Bayer, M., von Maurice, J., Ditton, H., & Blossfeld, H.-P. (2019). Measuring motivational concepts and personality aspects in the National Educational Panel Study. In H.-P. Blossfeld & H.-G. Roßbach (Eds.), Education as a lifelong process: The German National Educational Panel Study (NEPS) (2nd ed., pp. 155–169). Springer VS. DOI: 10.1007/978-3-658-23162-0_9

[119] Ziesmer, J. (2022). Samples, weights, and nonresponse: Wave 17 of the student sample of the National Educational Panel Study (supplement to NEPS:SC5:17.0.0). Bamberg, Germany: Leibniz Institute for Educational Trajectories, National Educational Panel Study. https://www.neps-data.de/Portals/0/NEPS/Datenzentrum/Forschungsdaten/SC5/17-0-0/SC5_17-0-0_W.pdf

[120] Zinn, S., Steinhauer, H. W., & Aßmann, C. (2017). Samples, weights, and nonresponse: The student sample of the National Educational Panel Study (wave 1 to 8) (NEPS Survey Paper No. 18). Bamberg, Germany: Leibniz Institute for Educational Trajectories, National Educational Panel Study. DOI: 10.5157/NEPS:SP18:1.0

[121] Zinn, S., Würbach, A., Steinhauer, H. W., & Hammon, A. (2018). Attrition and selectivity of the NEPS Starting Cohorts: An overview of the past 8 years (NEPS Survey Paper No. 34). Bamberg, Germany: Leibniz Institute for Educational Trajectories, National Educational Panel Study. DOI: 10.5157/NEPS:SP34:1.0

