# Supplementary material for: The German Panel of Teacher Education Students: Surveying (Prospective) Teachers from Higher Education into Working Life
Source: J Open Psychol Data. 2023 May 10;11:7. doi: 10.5334/jopd.76 (PMC12270278; doi:10.5334/jopd.76)
Supplement: Appendix. — Tables A1 to A12. [file jopd-11-76-s1.pdf]

Appendix

Con-struct	Dimension/ subscale	Variable names[a)]	Measurement points (wave)	Target group[b)]	Source[c)]
Learning environment in higher education (study programme)	*Structure*:<br>– Coordination of courses	3 items: t24340*	2/6/8	Under-graduate and graduate students in higher education; measured once during the bachelor or state examination degree course and the master degree course	1
	– Structuredness of lectures and classes	4 items: t24341*			
	Support:<br>– Rapport with lecturers	3 items: t24440*	2/6/8/11/14/ 17/19		
	– Rapport with fellow students	3 items: t24441*			
	– Motivation	3 items: t24442*			
	Challenge:<br>– Pressure to perform	3 items: t24540*	2/6/8/11/14/ 17/19		
	– Meaning orientation	4 items: t24541*			
	– Knowledge construction	4 items: t24542*			
	– Reproduction orientation	2 items: t24543*	2/6/8		
	– Knowledge transmission	3 items: t24544*			
	Orientation:<br>– Research orientation	4 items: t24640*	2/6/8/11/14/ 17/19		
	– Practice orientation	3 items: t24641*			
	– Interdisciplinarity	3 items: t24642*			
Social and academic integration	*Academic integration*<br>– Affective involvement	3 items: tg53232, ~4, ~6	2/4/6/8	Under-graduate and graduate students in higher education	2
	– Achievement orientation	3 items: tg53231, ~3, 5	2/4/6/8		
	– Perceived academic performance (as compared with own expectations)	3 items: tg5321*	2/4/6/8/11/14/ 17/19		
	– Perceived academic performance (as compared with fellow students)	4 items: tg5204*	4/6/8/11/14/17 /19		
	Social integration<br>– Interaction with faculty	4 items: tg5311*	2/4/6/8		
	– Interaction with fellow students	3 items: tg5312*			
Dropout intentions		4 items: tg5322*	2/4/6/8/11/14/ 17/19	Under-graduate and graduate students in higher education	3

a) The asterisk (*) is used to refer to a range of variables starting with the specified prefix. The tilde (~) before one or two characters is a placeholder for all but the last or last two characters of the last-mentioned variable.
b) SP = study participants; c) The sources and related references are listed after Table A11.

Table A1 Overview of selected constructs measured in NEPS SC5

Con-struct	Dimension/ subscale	Variable names[1)]	Measurement points (wave)	Target group[b)]	Source[c)]
Learning moti-vation	General intrinsic motivation	4 items: t66405*	5	Under-grad-uate and graduate stu-dents in high-er education	4
	Performance-related extrinsic motivation	4 items: t66406*			
	Competition-related extrinsic motivation	4 items: t66407*			
	Career-related extrinsic motivation	4 items: t66408*			
Absolute acade-mic self-concept		4 items: t66007*	2/6	Under-grad-uate and graduate stu-dents in high-er education	5
Study-specific helpless ness		3 items: t66010*	2/6	Under-gra-duate and graduate students in higher edu-cation	6
Study satis-faction	Satisfaction with study content	3 items: tg2411a, ~d, ~g	3	Under-grad-uate students in higher edu-cation	7
	Satisfaction with study conditions	3 items: tg2411b, ~e, ~h			
	Satisfaction with coping with study burdens	3 items: tg2411c, ~f, ~i			
Social com-peten-cies	Negative asser-tiveness	5 items: t67809*	15	All SP	8
	Conflict management	5 items: t67810*			
Stress		11 items: t5270*	6/11	All SP	9
Satis-faction	CAWI	8 items: t51400*	2/4/6/8	All SP	10
	CATI (same wording except instruction)	7 items: t51400*	7/9/10/12/13/15/16/18/		
General interest orienta-tions	Realistic	3 items: t66207a, ~g, ~m	1/9/18	All SP	11
	Investigative	3 items: t66207b, ~h, ~n			
	Artistic	3 items: t66207c, ~I, ~o			
	Social	3 items: t66207d, ~j, ~p			
	Enterprising	3 items: t66207e, ~k, ~q			
	Conventional	3 items: t66207f, ~l, ~r			

a) The asterisk (*) is used to refer to a range of variables starting with the specified prefix. The tilde (~) before one or two characters is a placeholder for all but the last or last two characters of the last-mentioned variable. b) SP = study participants; c) The sources and related references are listed after Table A11.

Table A1 cont.

**Con-struct**	**Dimension/ subscale**	**Variable names**[1)]	**Measurement points (wave)**	**Target group**[b)]	**Source**[c)]
Meaning of work	Learning aspects	2 items: t66210a, ~b	3	All SP	12
	Social orientation	3 items: t66210l, ~f, ~p			
	Autonomy	2 items: t66210m, ~o			
	Economic aspects	3 items: t66210c, ~g, ~h			
	Comfort aspects	2 items: t66210d, ~j			
	Expressive aspects	4 items: t66210e, ~k, ~f, ~i			
Big Five persona-lity fac-tors I	Extraversion	2 items: t66800a, ~f	3/10	All SP	13
	Agreeableness	3 items: t66800b, ~g, ~k			
	Conscientiousness	2 items: t66800c, ~h			
	Neuroticism	2 items: t66800d, ~i			
	Openness	2 items: t66800e, ~j			
Big Five persona-lity fac-tors II	Extraversion	4 items: t66800a, ~f, ~n, ~p	19	All SP	14
	Agreeableness	4 items: t66800b, ~g, ~q, ~t			
	Conscientiousness	4 items: t66800c, ~h, ~r, ~u			
	Neuroticism	4 items: t66800d, ~l, ~l, ~s			
	Openness	5 items: t66800e, ~j, ~m, ~o, ~v			
Self-esteem		10 items: t66003*	3/10	All SP	15
Goal pursuit	Tenacious goal pursuit	5 items: t67001*	4/14	All SP	16
	Flexible goal adjustment	5 items: t67000*			
Locus of control	Internal	2 items: t67010a, ~c	16	All SP	17
	External	2 items: t67010b, ~d			
Job satis-faction		15 items: tg770*		SP in employment	18

a) The asterisk (*) is used to refer to a range of variables starting with the specified prefix. The tilde (~) before one or two characters is a placeholder for all but the last or last two characters of the last-mentioned variable. b) SP = study participants; c) The sources and related references are listed after Table A11.

Table A1 cont.

Con-struct	Dimension/ subscale	Variable names[1)]	Measurement points (wave)	Target group[b)]	Source[c)]
Job require-ments	Novelty	4 items: tg7820*	14/17/19	SP in employ-ment	
	Complexity<br>– Dynamics-reactive	3 items: tg7811*			19
	– Dynamics-proactive	3 items: tg7812*			
	– Variety	3 items: tg7813*			
	Autonomy<br>– Method	3 items: tg7831*			
	– Criteria	3 items: tg7832*			
	– Scheduling	3 items: tg7833*			
	Research involvement<br>– Consumption/ application	3 items: tg7841*			20
	– Active research	4 items: tg7842*			

a) The asterisk (*) is used to refer to a range of variables starting with the specified prefix. The tilde (~) before one or two characters is a placeholder for all but the last or last two characters of the last-mentioned variable. b) SP = study participants; c) The sources and related references are listed after Table A11.

Table A1 cont.

Construct	Dimension/ subscale	Variable names[a)]	Measurement points (wave)	Target group[b)]	Source[c)]
Basic information	Type of teaching track	tg6402*	9/10/12/13/15/16/18/19	SP with a completed or ongoing preparatory service; measured once	21
	Type of school	tg64033			
	Number of teaching hours per week	tg64041			
Learning environment	*Instrumental support* – mentor teacher	3 items: tg6411*	9/10/12/13/15/16/18/19	SP with a completed or ongoing preparatory service; measured once	22
	– head of teaching seminar	3 items: tg6421*			
	– fellow trainee teachers	3 items: tg6431*			
	Interaction (with mentor) – transmissive	3 items: tg64122, ~31, ~33			
	– constructivist	4 items: tg64121, ~23, ~32, ~34			
	Discourse and reflection (teaching seminar) – discussing different views	3 items: tg6422*			
	– integration of theory and practice	4 items: tg6423*			
Dropout intention		1 item: tg64051	9/10/12/13/15/16/18/19	SP in preparatory service; measured once	21
Teaching practices	Appropriate planning and implementation of instruction	6 items: tg6511*	9/10/12/13/15/16/18/19	SP in preparatory service, who are involved in teaching; measured once	23
	Designing supporting, motivating, and transfer-oriented learning situations	8 items: tg6512*			

a) The asterisk (*) is used to refer to a range of variables starting with the specified prefix. The tilde (~) before one or two characters is a placeholder for all but the last or last two characters of the last-mentioned variable. b) SP = study participants; c) The sources and related references are listed after Table A11.

Table A2 Overview of constructs measured with respect to the preparatory service

Construct	Dimension/ subscale	Variable names[a)]	Measurement points (wave)	Target group[b)]	Source[c)]
Learning opportunities: inclusion, multiculturalism		5 items: tg6011* (reduced to 1 item in wave 19)	11/12/14/17/ 19	SP in a teaching degree programme, with a degree in teaching or a second state examination, (trainee) teachers	24
Professional experiences: inclusion, multiculturalism		2 items: tg6012* (reduced to 1 item in wave 19)	11/12/14/17/ 19	SP in a teaching degree programme, with a degree in teaching or a second state examination, (trainee) teachers	25
Professional experiences: special educational needs		8 items: tg6013*	14/17/19	SP in a teaching degree programme, with a degree in teaching or a second state examination, (trainee) teachers	26
Professional experiences: digital media in the classroom		1 item: tg84411	17/19	SP with a completed or ongoing prepara-tory service or work-ing as teachers	25
Impediment of teaching	Cultural and social heterogeneity	3 items: tg60291 to tg60293	16/18/19	SP working as teachers	27
	Performance heterogeneity	3 items: tg60294 to tg60296			
	Behaviour/ motivation	3 items: tg60297 to tg60299			

a) The asterisk (*) is used to refer to a range of variables starting with the specified prefix. The tilde (~) before one or two characters is a placeholder for all but the last or last two characters of the last-mentioned variable. b) SP = study participants; c) The sources and related references are listed after Table A11.

Table A3 Overview of constructs measuring learning opportunities and professional experiences

Construct	Dimension/ subscale	Variable names[a)]	Measurement points (wave)	Target group[b)]	Source[c)]
Motivation for choosing teacher education	Educational interest	3 items: tg6103* or tg6113*	8	SP in a teaching degree programme (tg610*) or with a degree in teaching (tg611*)	28
	Subject-specific interest	3 items: tg6106* or tg6116*			
	Time for family/ leisure	3 items: tg6102* or tg6112*			
	Financial security	3 items: tg6101* or tg6111*			
	Ability beliefs	3 items: tg6104* or tg6114*			
	Low-difficulty degree programme	3 items: tg6107* or tg6117*			
	Social influences	3 items: tg6105* or tg6115*			
Motivation for choosing teaching as a career	Educational interest	3 items: tg6121*	19	Teachers without a higher education degree in teaching	29
	Subject-specific interest	3 items: tg6122*			
	Time for family/ leisure	3 items: tg6123*			
	Financial security	3 items: tg6124*			
	Fallback career	3 items: tg6125*			
Teacher enthusiasm		4 items: tg6941*	11/14	SP in a teaching degree programme, with a degree in teaching or a second state examination, (trainee) teachers	30
General teacher self-efficacy		10 items: tg6800*	9/10/12/13/ 15/16/18/19	SP in a teaching degree programme, with a degree in teaching or a second state examination, (trainee) teachers	31

a) The asterisk (*) is used to refer to a range of variables starting with the specified prefix. The tilde (~) before one or two characters is a placeholder for all but the last or last two characters of the last-mentioned variable. b) SP = study participants; c) The sources and related references are listed after Table A11.

Table A4 Overview of constructs measuring general aspects of professional competencies

Construct	Dimension/ subscale	Variable names[a)]	Measurement points (wave)	Target group[b)]	Source[c)]
Beliefs about teaching & learning I	Direct transmission beliefs	4 items: tg6201*	8	SP in a teaching degree programme or with a degree in teaching	32
	Constructivist beliefs	4 items: tg6202*			
Beliefs about teaching & learning II	Transmission beliefs	4 items: tg6911*	11/14/17/19	SP in a teaching degree programme, with a degree in teaching or a second state examination, (trainee) teachers	30
	Constructivist beliefs	4 items: tg6912*			
Teachers' professional self-concept	Subject	3 items: tg6301*	8 (all four scales) 11/14/17/19 (without subscale "consulting")	SP in a teaching degree programme, with a degree in teaching or a sec-ond state exami-nation, (trainee) teachers	33
	Education	3 items: tg6302*			
	Diagnostics	3 items: tg6303*			
	Consulting	3 items: tg6304*			
Occupa-tional self-regulation	Subjective importance of work	3 items: tg6951*	11/14/17/19	(Trainee) teachers	34
	Work-related ambition	4 items: tg6952*			
	Emotional distancing	3 items: tg6961*			
	Tendency to resignation in the face of failure	3 items: tg6962*			

a) The asterisk (*) is used to refer to a range of variables starting with the specified prefix. The tilde (~) before one or two characters is a placeholder for all but the last or last two characters of the last-mentioned variable. b) SP = study participants; c) The sources and related references are listed after Table A11.

Table A4 cont.

Construct	Dimension/ subscale	Variable names[a)]	Measurement points (wave)	Target group[b)]	Source[c)]
Self-efficacy regarding inclusive education		4 items: tg6942*	11/14/17	SP in a teaching degree programme, with a degree in teaching or a second state examination, (trainee) teachers	35
Self-efficacy regarding teaching in culturally diverse classes		3 items: tg6943*	11/14/17/19	SP in a teaching degree programme, with a degree in teaching or a second state examination, (trainee) teachers	36
Self-efficacy regarding teaching with digital media		5 items: tg8311*	17/19	SP with a completed or ongoing preparatory service or working as teachers	37
Beliefs about inclusion	Beliefs about the arrangement of inclusive education	3 items: tg6921*	11/14/17	SP in a teaching degree programme, with a degree in teaching or a second state examination, (trainee) teachers	35
	Beliefs about the effects of inclusive learning	3 items: tg69221, ~2, ~3			
Cultural beliefs	Multicultural beliefs	3 items: tg6931*	11/14/17/19	SP in a teaching degree programme, with a degree in teaching or a second state examination, (trainee) teachers	38
	Egalitarian beliefs	3 items: tg6932*			
	Assimilative beliefs	3 items: tg6933*	14/17/19		

a) The asterisk (*) is used to refer to a range of variables starting with the specified prefix. The tilde (~) before one or two characters is a placeholder for all but the last or last two characters of the last-mentioned variable. b) SP = study participants; c) The sources and related references are listed after Table A11.

Table A5 Overview of constructs measuring specific aspects of professional competencies (dealing with inclusive education, cultural diversity, and digital media, teachers' stereotypes)

Construct	Dimension/ subscale	Variable names[a)]	Measurement points (wave)	Target group[b)]	Source[c)]
Beliefs about the effects of ICT use	Positive effects	6 items: tg8711*	19	SP with a completed or ongoing prepa-ratory service or working as teachers	39
	Negative effects	4 items: tg8721*			
ICT-related ability self-concept		7 items: tg8511*	19	SP with a completed or ongoing prepa-ratory service or working as teachers	39
Teachers' stereotypes	Stereotypes regarding reading competence of groups with dif-ferent social backgrounds	3 items: t31602a, t31602b, t31602c	14/17	SP in a teaching degree programme, with a degree in teaching or a second state examination, (trainee) teachers	40
	Stereotypes regarding reading competence of girls and boys	2 items: t31602d, t31602e			
	Stereotypes regarding reading competence of immigrants and ethnic majority students	4 items: t31602f, t31602g, t31602h, t31602i			
	Stereotypes regarding parental support of groups with different social backgrounds	3 items: t31603a, t31603b, t31603c			
	Stereotypes regarding parental support of girls and boys	2 items: t31603d, t31603e			
	Stereotypes regarding parental support of immigrants and ethnic majority students	4 items: t31603f, t31603g, t31603h, t31603i			

a) The asterisk (*) is used to refer to a range of variables starting with the specified prefix. The tilde (~) before one or two characters is a placeholder for all but the last or last two characters of the last-mentioned variable. b) SP = study participants; c) The sources and related references are listed after Table A11.

Table A5 cont.

Construct	Dimension/ subscale	Variable names[a)]	Measurement points (wave)	Target group[b)]	Source[c)]
School type		1 item: tg60211 (CAWI); tg60212 (CATI)	11/12/13/14/ 15/16/17/18/ 19	SP working as teachers (CATI) or (trainee) teachers (CAWI)	41
Length of employment at current school		1 item: tg60251	14/17/19	SP working as (trainee) teachers	42
Percentage of migrants at school		1 item: tg60261 (CAWI); tg60262 (CATI)	14/15/16/17/ 18/19	SP working as teachers	26
Position as school principal		1 item: tg60029 (CAWI); tg60020 (CATI)	14/15/16/17/ 18/19	SP working as teachers	43
Subjects taught		10 items: tg6022* (CAWI); tg6024* (CATI)	11/12/13/14/ 15/16/17/18/ 19	SP working as teachers (CATI) or (trainee) teachers (CAWI)	41
Classes taught		1 item: tg60231 (CAWI); tg60232 (CATI)	11/12/13/14/ 15/16/17/18/ 19	SP working as teachers (CATI) or (trainee) teachers (CAWI)	41
Class context/ class composition		4 items: tg6028*	16/18/19	SP working as teachers	44

a) The asterisk (*) is used to refer to a range of variables starting with the specified prefix. The tilde (~) before one or two characters is a placeholder for all but the last or last two characters of the last-mentioned variable. b) SP = study participants; c) The sources and related references are listed after Table A11.

Table A6 Overview of constructs measuring the professional situation

Construct	Dimension/ subscale	Variable names[a)]	Measurement points (wave)	Target group[b)]	Source[c)]
Cognitive activation		3 or 5 items: tg6991*	3 items: 11<br>5 items: 14/17/19	SP working as (trainee) teachers	30
Classroom management	Disruptions/ effective use of time	3 items: tg6971*	11/14/17/19	SP working as (trainee) teachers	45
	Monitoring	3 or 5 items: tg69721, ~2, ~3, ~4, tg69225	3 items: 11<br>5 items: 14/ 17/19		
Differentiation/ individualisation		6 items: tg6981*	11/14/17/19	SP working as (trainee) teachers	46
Emphasis on devel-oping students' digital and ICT competencies		9 items: tg8611*	19	SP working as (trainee) teachers	39
Proactive behaviour		5 items: tg6041*	19	SP working as teachers or in other professions	30

a) The asterisk (*) is used to refer to a range of variables starting with the specified prefix. The tilde (~) before one or two characters is a placeholder for all but the last or last two characters of the last-mentioned variable. b) SP = study participants; c) The sources and related references are listed after Table A11.

Table A7 Overview of constructs measuring teaching practices and proactive behaviour

Construct	Dimension/ subscale	Variable names[a)]	Measurement points (wave)	Target group[b)]	Source[c)]
Teaching situation during pandemic		2 items: tg8400*	17/19	SP working as (trainee) teachers	47
Context of teaching during pandemic	School type, grades, teaching subject	3 items: tg8410*	17/19	SP working as (trainee) teachers	
Provision of learning materials	Communication ways with students and parents	9 items: tg8421*	17/19	SP working as (trainee) teachers	
	Most frequently used commu-nication way	1 item: tg84221			
Consequences for preparatory service		8 items: tg8451*	17/19	SP in preparatory service	
Occupational challenges during pandemic		5 items: tg8431*	17/19	SP working as (trainee) teachers who taught last school term (prior to survey)	48
Use of digital media for teaching		5 items: tg8442*	17/19	SP working as (trainee) teachers who taught last school term (prior to survey)	49

a) The asterisk (*) is used to refer to a range of variables starting with the specified prefix. The tilde (~) before one or two characters is a placeholder for all but the last or last two characters of the last-mentioned variable. b) SP = study participants; c) The sources and related references are listed after Table A11.

Table A8 Overview of constructs measuring the experiences and situation during the Corona pandemic

Construct	Dimension/ subscale	Variable names[a)]	Measurement points (wave)	Target group[b)]	Source[c)]
Further education (screening)		1 item: tg60311	15/16/18/19	SP working as teachers who participated in at least one further training	21
Further education topics		11 items: tg6034*	15/16/18/19	SP working as teachers if tg60311==1 (yes)	50
Subjective importance of further education		5 Items: tg6033*	15/16/18/19	SP working as teachers	51
Climate for further education at school		7 Items: tg6032*	15/16/18/19	SP working as teachers	52

a) The asterisk (*) is used to refer to a range of variables starting with the specified prefix. The tilde (~) before one or two characters is a placeholder for all but the last or last two characters of the last-mentioned variable. b) SP = study participants; c) The sources and related references are listed after Table A11.

Table A9 Overview of constructs measuring (predictors of) participation in professional development activities

Construct	Dimension/ subscale	Variable names[a)]	Measurement points (wave)	Target group[b)]	Source[c)]
Teacher cooperation	Exchange	3 items: tg8111*	14/17/19	SP working as teachers	53
	Joint work	3 items: tg8112*			
	Co-construction	4 items: tg8113*			
Instructional leadership		4 or 5 items: tg8220*	4 items: 14<br>5 items: 17/19	SP working as teachers but not as school principals	54
Transformational leadership	Articulating a vision	3 items: tg8211*	14/17/19	SP working as teachers but not as school principals	55
	Fostering the acceptance of group goals	3 items: tg8212*			
	Providing an appropriate model	3 items: tg8213*			

a) The asterisk (*) is used to refer to a range of variables starting with the specified prefix. The tilde (~) before one or two characters is a placeholder for all but the last or last two characters of the last-mentioned variable. b) SP = study participants; c) The sources and related references are listed after Table A11.

Table A10 Overview of constructs measuring teacher cooperation and leadership styles of school principals

Construct	Dimension/ subscale	Variable names[a)]	Measurement points (wave)	Target group[b)]	Source[c)]
Emotional exhaustion		4 Items: tg6600*	9/10/12/13/15/ 16/18/19	SP with a completed or ongoing preparatory service or working as teachers	30
Job satis- faction		4 items: tg6700*	9/10/12/13/15/ 16/18/19	SP with a completed or ongoing preparatory service or working as teachers	30

a) The asterisk (*) is used to refer to a range of variables starting with the specified prefix. The tilde (~) before one or two characters is a placeholder for all but the last or last two characters of the last-mentioned variable. b) SP = study participants; c) The sources and related references are listed after Table A11.

Table A11 Overview of constructs measuring emotional exhaustion and job satisfaction

Sources of the survey instruments described in Tables A1 to A11

1 Cf. Schaeper & Weiß, 2016
2 Cf. Dahm et al., 2016; Schaeper, 2020
3 Trautwein et al., 2006
4 Schiefele et al., 2002
5 Dickhäuser et al., 2002
6 Jerusalem & Schwarzer, 2008
7 Selected from Westermann et al., 1996
8 Adapted from Riemann & Allgöwer, 1993
9 Cf. Gross & Seebaß, 2016
10 Cf. Bela et al., 2018
11 Cf. Wohlkinger et al., 2019
12 Taken or adapted from Borchert & Landherr, 2007; Trautwein et al., 2006
13 Rammstedt & John, 2007
14 Rammstedt & John, 2005
15 Collani & Herzberg, 2003
16 Short version of Brandtstädter & Renner, 1990
17 Kovaleva et al., 2014
18 Adapted from Fabian et al., 2013; Hall, 2009; Neuberger & Allerbeck, 1978
19 Trommer et al., 2021
20 Briedis et al., 2016
21 Own development
22 Adapted from Kunter et al., 2017; Max-Planck-Institut für Bildungsforschung, 2010
23 Weresch-Deperrois et al., 2009
24 Laschke & König, 2014
25 Stiftung Universität Hildesheim, 2016
26 NEPS SC3/SC4 (NEPS, 2012)
27 Baumert et al., 2008
28 Selected scales and items taken from Pohlmann & Möller, 2010; Retelsdorf & Möller, 2012
29 Adapted from Pohlmann & Möller, 2010; Retelsdorf & Möller, 2012; fallback career: Watt et al., 2012
30 Adapted from Kunter et al., 2017
31 Schwarzer & Schmitz, 1999
32 OECD, 2009
33 Adapted from Retelsdorf et al., 2014
34 Adapted from Schaarschmidt & Fischer, 2001; cf. Menge & Schaeper, 2019

35 Adapted from Bosse & Spörer, 2014
36 Adapted from Max-Planck-Institut für Bildungsforschung, 2010
37 Adapted from Bosse & Spörer, 2014; Dinse de Salas, 2019; and own development
38 Adapted from Hachfeld et al., 2011; subscale "assimilation" developed by A. Hachfeld and NEPS, Pillar 4
39 Adapted from Vennemann et al., 2021
40 Wenz, 2020; Wenz et al., 2016
41 Adapted from Brachem, 2016
42 Adapted from Artelt et al., 2013
43 Adapted from Paulus et al., 2016
44 Adapted from Helmke et al., 2000; Max-Planck-Institut für Bildungsforschung, 2010; NEPS SC3/SC4 (NEPS, 2012)
45 Adapted or taken from Kunter et al., 2017; Thiel et al., 2012; Wettstein et al., 2017
46 Adapted from Baumert et al., 2008
47 Own development, partly adapted from Brachem (2016), Lorenz et al. (2020), Bos et al. (2010) and the NEPS Corona Module (NEPS-C)
48 Adapted from Lorenz et al., 2020
49 Adapted from Bos et al., 2010
50 Adapted from NEPS SC4 (LIfBi, 2016); Europäische Kommission/EACEA/Eurydice, 2015; Gagarina & Saldern, 2010; Hußmann et al., 2017; own development
51 Richter et al., 2014
52 Richter et al., 2014; adapted from Bos et al., 2005
53 Dizinger & Fussangel, 2008; see also Dizinger, 2015
54 Adapted from Pietsch et al., 2014
55 Adapted from Ewen, 2013

Sources: References

Artelt, C., Blossfeld, H.-P., Faust, G., Roßbach, H.-G., & Weinert, S. (2013). *Bildungsprozesse, Kompetenzentwicklung und Selektionsentscheidungen im Vorschul- und Schulalter (BiKS-8-14) – Codebuch zum Lehrerfragebogen Welle 3* [Educational processes, competence development and selection decisions in preschool and school age – Codebook for the teacher questionnaire wave 3]. Berlin, Germany. IQB – Institut zur Qualitätsentwicklung im Bildungswesen. https://www.iqb.hu-berlin.de/fdz/studies/BiKS_8-14/Lehrerfragebogen_2.pdf

Baumert, J., Blum, W., Brunner, M., Dubberke, T., Jordan, A., Klusmann, U., Krauss, S., Kunter, M., Löwen, K., Neubrand, M., & Tsai, Y.-M. (2008). *Professionswissen von Lehrkräften, kognitiv aktivierender Mathematikunterricht und die Entwicklung von mathematischer Kompetenz (COACTIV): Dokumentation der Erhebungsinstrumente* [Professional competence of teachers, cognitively activating instruction, and the development of students' mathematical literacy (COACTIV): Documentation of the

survey instruments]. *Materialien aus der Bildungsforschung: Vol. 83*. Max-Planck-Institut für Bildungsforschung. https://pure.mpg.de/pubman/item/item_2100057_8/component/file_2197666/Materialien_Bildungsforschung_MPIB_083.pdf

Bela, A., Feinauer, N., Gebel, M., Heineck, G., Lettau, J., & Mergard, K. (2018). *Returns to education across the life course in the National Educational Panel Study: Theoretical framework and corresponding survey program*. (NEPS Survey Paper No. 50). Bamberg, Germany. Leibniz Institute for Educational Trajectories. https://doi.org/10.5157/NEPS:SP50:1.0

Borchert, M., & Landherr, G. (2007). *Meaning of Work. Auswertung einer Befragung von Teilnehmern des Management Forum Starnberg, durchgeführt im November 2006*. Unpublished manuscript [Meaning of Work. Analysis of a survey of participants in the Management Forum Starnberg, conducted in November 2006]. Duisburg and Essen, Germany. Universität Duisburg-Essen.

Bos, W., Lankes, E.-M., Prenzel, M., Schwippert, K., Valtin, R., Voss, A., & Walther, G. (Eds.). (2005). *IGLU: Skalenhandbuch zur Dokumentation der Erhebungsinstrumente* [IGLU: Scale manual for the documentation of the survey instruments]. Waxmann.

Bos, W., Strietholt, R., Goy, M., Stubbe, T. C., Tarelli, I., & Hornberg, S. (2010). *IGLU 2006. Dokumentation der Erhebungsinstrumente* [IGLU 2006. Documentation of survey instruments]. Waxmann.

Bosse, S., & Spörer, N. (2014). Erfassung der Einstellung und der Selbstwirksamkeit von Lehramtsstudierenden zum inklusiven Unterricht [Assessment of attitudes and self-efficacy of pre-service teachers towards inclusive education]. *Empirische Sonderpädagogik, 6*(4), 279–299.

Brachem, J.-C. (2016). *Erklärung der Resignationstendenz von Lehrkräften durch individuelle Tätigkeits- und Kompetenzprofile und bildungspolitische Rahmenbedingungen* [Explaining teachers' resignative tendencies through individual activity and competency profiles and education policy conditions] [Dissertation]. Carl von Ossietzsky Universität, Oldenburg. http://oops.uni-oldenburg.de/2866/1/braerk16.pdf

Brandtstädter, J., & Renner, G. (1990). Tenacious goal pursuit and flexible goal adjustment: Explication and age-related analysis of assimilative and accommodative strategies of coping. *Psychology and Aging*, *5*(1), 58–67. https://doi.org/10.1037//0882-7974.5.1.58

Briedis, K., Klüver, S., & Trommer, M. (2016). *Zwischen Etablierung, Stabilisierung und Aufstieg: Berufliche Entwicklung der Hochschulabsolvent(inn)en 2009* [Between establishment, stabilisation and advancement: Professional development of higher education graduates 2009]. *Forum Hochschule: 4/2016*. DZHW. https://www.dzhw.eu/pdf/pub_fh/fh-201604.pdf

Collani, G. von, & Herzberg, P. Y. (2003). Eine revidierte Fassung der deutschsprachigen Skala zum Selbstwertgefühl von Rosenberg [A revised version of the German adaptation of Rosenberg's self-esteem scale]. *Zeitschrift für Differentielle und Diagnostische Psychologie*, *24*(1), 3–7. https://doi.org/10.1024//0170-1789.24.1.3

Dahm, G., Lauterbauch, O., & Hahn, S. (2016). Measuring students' social and academic integration – assessment of the operationalization in the National Educational Panel Study. In H.-P. Blossfeld, J. von Maurice, M. Bayer, & J. Skopek (Eds.), *Methodological issues of longitudinal surveys: The example of the National*

Educational Panel Study (pp. 313–329). Springer VS. https://doi.org/10.1007/978-3-658-11994-2_18

Dinse de Salas, S. (2019). *Digitale Medien im Unterricht – Entwicklung professionellen Wissens und professionsbezogener Einstellungen durch Coaching* [Digital media in the classroom – Development of professional knowledge and profession-related attitudes through coaching] [Dissertation]. Pädagogische Hochschule Heidelberg, Heidelberg. https://opus.ph-heidelberg.de/files/335/Dissertation_Dinse_de_Salas.pdf

Gagarina, L., & Saldern, M. von. (2010). Professionalisierung der Lehrkräfte [Professionalisation of teachers]. In M. Demmer & M. von Saldern (Eds.), "*Helden des Alltags". Erste Ergebnisse der Schulleitungs- und Lehrkräftebefragung (TALIS) in Deutschland* (pp. 47–63). Waxmann.

Gross, C., & Seebaß, K. (2016). The Standard Stress Scale (SSS): Meauring stress in the life course. In H.-P. Blossfeld, J. von Maurice, M. Bayer, & J. Skopek (Eds.), *Methodological issues of longitudinal surveys: The example of the National Educational Panel Study* (pp. 233–249). Springer VS. https://doi.org/10.1007/978-3-658-11994-2_14

Dickhäuser, O., Schöne, C., Spinath, B., & Stiensmeier-Pelster, J. (2002). Die Skalen zum akademischen Selbstkonzept: Konstruktion und Überprüfung eines neuen Instruments [The academic self concept scales: Construction and evaluation of a new instrument]. *Zeitschrift für Differentielle und Diagnostische Psychologie*, *23*(4), 393–405. https://doi.org/10.1024//0170-1789.23.4.393

Dizinger, V. (2015). *Professionelle und interprofessionelle Kooperation von Lehrerinnen und Lehrern im Kontext schulischer Belastung und Beanspruchung* [Professional and interprofessional cooperation of teachers in the context of school stress and strain] [Dissertation]. Universität Bielefeld, Bielefeld. https://pub.uni-bielefeld.de/download/2730533/2730536

Dizinger, V., & Fussangel, K. (2008). *Skalenhandbuch zum Projekt "Kooperationsformen und Beanspruchungserleben an Halb- und Ganztagsschulen".* Unpublished manuscript [Documentation of survey instruments for the project "Forms of cooperation and experienced stress at half-day and all-day schools"]. Wuppertal, Germany. Universität Wuppertal.

Europäische Kommission/EACEA/Eurydice. (2015). *Der Lehrerberuf in Europa: Praxis, Wahrnehmungen und politische Maßnahmen* [The teaching profession in Europe: Practices, perceptions, and policies]. *Eurydice-Bericht*. Amt für Veröffentlichungen der Europäischen Union.

Ewen, C. P. J. (2013). *Politische Fertigkeiten im Führungskontext.* [Political skill in leadership context] [Dissertation]. Universität Bonn, Bonn. http://hss.ulb.uni-bonn.de/2013/3115/3115.pdf

Fabian, G., Rehn, T., Brandt, G., & Briedis, K. (2013). *Karriere mit Hochschulabschluss? Hochschulabsolventinnen und -absolventen des Prüfungsjahrgangs 2001 zehn Jahre nach dem Studienabschluss*. [A career with a degree? Higher education graduates of the 2001 examination year ten years after graduation]. *Forum Hochschule: 10|2013*. HIS. https://www.dzhw.eu/pdf/pub_fh/fh-201310.pdf

Hachfeld, A., Hahn, A., Schroeder, S., Anders, Y., Stanat, P., & Kunter, M. (2011). Assessing teachers' multicultural and egalitarian beliefs: The Teacher Cultural Beliefs Scale. *Teaching and Teacher Education*, *27*(6), 986–996. https://doi.org/10.1016/j.tate.2011.04.006

Hall, A. (2009). *Die BIBB/BAuA-Erwerbstätigenbefragung 2006: Methodik und Frageprogramm im Vergleich zur BIBB/IAB-Erhebung 1998* [BIBB/BAuA Employment Survey 2006: Methodology and survey programme in comparison with the BIBB/IAB survey 1998]. *Wissenschaftliche Diskussionspapiere: Vol. 107*. Bundesinstitut für Berufsbildung. http://www.bibb.de/dokumente/pdf/wd_107_bibb_baua_erwerbstaetigenbefragung_2006.pdf

Helmke, A., Ridder, A., & Schrader, F.-W. (2000). *Projekt MARKUS (Mathematik-Gesamterhebung Rheinland-Pfalz: Kompetenzen, Unterrichtsbedingungen, Schulkontext). Fragebogen für Lehrerinnen und Lehrer* [Project MARKUS (Mathematics full survey Rhineland-Palatinate: Competencies, teaching conditions, school context). Questionnaire for teachers]. Landau, Germany. Fachbereich Psychologie der Universität Koblenz-Landau, Abt. Landau. http://andreas-helmke.de/wordpress/wp-content/uploads/2015/01/markus_LehrerFB.pdf

Hußmann, A., Wendt, H., Bos, W., Bremerich-Vos, A., Kasper, D., Lankes, E.-M., McElvany, N., Stubbe, T. C., & Valtin, R. (Eds.) (2017). *IGLU 2016: Lesekompetenzen von Grundschulkindern in Deutschland im internationalen Vergleich* [IGLU 2016: Reading competencies of primary school children in Germany in international comparison]. Waxmann.

Jerusalem, M., & Schwarzer, R. (2008). Dimensionen der Hilflosigkeit [Dimensions of helplessness]. In A. Glöckner-Rist (Ed.), *Zusammenstellung sozialwissenschaftlicher Items und Skalen. ZIS Version 12.00.* GESIS.

Kovaleva, A., Beierlein, C., Kemper, C. J., & Rammstedt, B. (2014). Internale-Externale-Kontrollüberrzeugung-4 (IE-4) [Internal-external locus of control scale-4 (IE-4)]. *Zusammenstellung Sozialwissenschaftlicher Items und Skalen (ZIS)*. https://doi.org/10.6102/zis184

Kunter, M., Baumert, J., Leutner, D., Terhart, E., Seidel, T., Dicke, T., Kunina-Habenicht, O., Linninger, C., Lohse-Bossenz, H., Schulze-Stocker, F., & Stürmer, K. (2017). *Dokumentation der Erhebungsinstrumente der Projektphasen des BilWiss-Forschungsprogramms von 2009 bis 2016* [Documentation of the survey instruments of the BilWIss research programme 2009 to 2016]. Frankfurt a. M. IQB. https://www.iqb.hu-berlin.de/fdz/studies/BilWiss/BilWiss_Skalenha.pdf

Kunter, M., Baumert, J., Leutner, D., Terhart, E., Seidel, T., Dicke, T., Kunina-Habenicht, O., Linninger, C., Lohse-Bossenz, H., Schulze-Stocker, F., & Stürmer, K. (2017). *Dokumentation der Erhebungsinstrumente der Projektphasen des BilWiss-Forschungsprogramms von 2009 bis 2016* [Documentation of the survey instruments of the BilWIss research programme 2009 to 2016]. Frankfurt a. M. IQB. https://www.iqb.hu-berlin.de/fdz/studies/BilWiss/BilWiss_Skalenha.pdf

Laschke, C., & König, J. (2014). Erfassung der Lerngelegenheiten (opportunities to learn, OTL) [Measuring learning opportunities]. In C. Laschke & S. Blömeke (Eds.), *Teacher Education and Development Study: Learning to teach mathematics (TEDS-M 2008). Dokumentation der Erhebungsinstrumente* (S. 45–108). Waxmann.

LIfBi (2016). *Starting Cohort 4: Grade 9 (SC4). Waves 3 and 4. Questionnaires (SUF Version 4.0.0).* Bamberg, Germany. Leibniz Institute for Educational Trajectories (LIfBi). https://www.neps-data.de/Portals/0/NEPS/Datenzentrum/Forschungsdaten/SC4/4-0-0/SC4_4-0-0_en.pdf

Lorenz, R., Lepper, C., Brüggemann, T., & McElvany, N. (2020). *Unterricht während der Corona-Pandemie. Lehrkräftebefragung. Ergebnisse, Teil I "Der Unterricht"* [Teaching

during the Corona pandemic. Survey of teachers. Results, part I "Instruction"]. Dortmund, Germany. Institut für Schulentwicklungsforschung (IFS), Universität Dortmund. https://www.tu-dortmund.de/storages/zentraler_bilderpool/user_upload/UCP_Kurzbericht_final.pdf

Max-Planck-Institut für Bildungsforschung. (2010). *COACTIV-R: Eine Studie zum Erwerb professioneller Kompetenz von Lehramtsanwärtern während des Vorbereitungsdienstes – Dokumentation der Erhebungsinstrumente für den ersten und zweiten Messzeitpunkt.* Unpublished manuscript [COACTIV-R: A study on the acquisition of professional competence of teacher candidates during the induction phase – documentation of the survey instruments for the first and second measurement time point]. Berlin, Germany. Max-Planck-Institut für Bildungsforschung.

Menge, C., & Schaeper, H. (2019). Berufliche Selbstregulation von Lehrkräften: Überprüfung eines Kurzinstruments [Teachers' occupational self-regulation: Validation of a short measurement instrument]. *Zeitschrift für Erziehungswissenschaft*, *22*(6), 1489–1513. https://doi.org/10.1007/s11618-018-0851-x

NEPS (2012). *NEPS Starting Cohort 3: 5th grade (SC3)/NEPS Starting Cohort 4: 9th grade (SC4). Main study 2010/11, class teachers. PAPI questionnaire with variables (ID34).* University of Bamberg, National Educational Panel Study (NEPS). https://www.neps-data.de/Portals/0/NEPS/Datenzentrum/Forschungsdaten/SC4/1-0-0/MDE34_A28_A46_EC_en.pdf

Neuberger, O., & Allerbeck, M. (1978). *Messung und Analyse von Arbeitszufriedenheit: Erfahrungen mit dem "Arbeitsbeschreibungsbogen (ABB)"* [Measurement and analysis of job satisfaction: Experiences with the Job Description Questionnaire (ABB)"]. Huber.

OECD (Ed.). (2009). *Creating effective teaching and learning environments: First results from TALIS*. OECD.

Paulus, P., Horstmann, D., Baydar, C., & Dadaczynski, K. (2016). *Erster Bericht zur Online-Befragung "Mehr Zeit für gute Schule"* [First report on the online survey "More Time for Good Schools"]. Lüneburg, Germany. Zentrum für Angewandte Gesundheitswissenschaften (ZAG), Leuphana Universität Lüneburg. https://www.leuphana.de/fileadmin/user_upload/portale/ZAG_Schulbefragung-Niedersachsen/Mehr_Zeit_fuer_gute_Schule_Erster_Bericht_August_2016.pdf

Pietsch, M., Scholand, B., Graw, S., Hengstmann, E., & Kulin, S. (2014). *Skalenhandbuch der Schulinspektion Hamburg: Fragebögen für Pädagoginnen und Pädagogen, Eltern und Schülerinnen und Schüler (gültig ab 15.11.2013)* [Scale handbook of the Hamburg school inspection: Questionnaires for teachers, parents and students (valid from 15.11.2013)]. Hamburg, Germany. Institut für Bildungsmonitoring und Qualitätsentwicklung (ifbq).

Pohlmann, B., & Möller, J. (2010). Fragebogen zur Erfassung der Motivation für die Wahl des Lehramtsstudiums (FEMOLA) [Motivation for choosing teacher education questionnaire]. *Zeitschrift für Pädagogische Psychologie*, *24*(1), 73–84. https://doi.org/10.1024/1010-0652/a000005

Rammstedt, B., & John, O. P. (2005). Kurzversion des Big Five Inventory (BFI-K): Entwicklung und Validierung eines ökonomischen Inventars zur Erfassung der fünf Faktoren der Persönlichkeit [Short version of the Big Five Inventory (BFI-K): Development and validation of an economic inventory for assessment of the five factors of

personality]. *Diagnostica*, *51*(4), 195–206. https://doi.org/10.1026/0012-1924.51.4.195

Rammstedt, B., & John, O. P. (2007). Measuring personality in one minute or less: A 10-item short version of the Big Five Inventory in English and German. *Journal of Research in Personality*, *41*(1), 203–212. https://doi.org/10.1016/j.jrp.2006.02.001

Retelsdorf, J., Bauer, J., Gebauer, S. K., Kauper, T., & Möller, J. (2014). Erfassung berufsbezogener Selbstkonzepte von angehenden Lehrkräften (ERBSE-L) [Measuring prospective teachers' professional self-concept]. *Diagnostica*, *60*(2), 98–110. https://doi.org/10.1026/0012-1924/a000108

Retelsdorf, J., & Möller, J. (2012). Grundschule oder Gymnasium? Zur Motivation ein Lehramt zu studieren [Primary or secondary school? On the motivation for choosing teacher education]. *Zeitschrift für Pädagogische Psychologie, 26*(1), 5–17. https://doi.org/10.1024/1010-0652/a000056

Richter, D., Böhme, K., Bastian-Wurzel, J., Anand Pant, H., & Stanat, P. (2014). *IQB-Ländervergleich 2011: Skalenhandbuch zur Dokumentation der Erhebungsinstrumente* [IQB National Assessment Study 2011 scaling manual. Documentation of the survey instruments]. Humboldt-Universität zu Berlin, Institut zur Qualitätsentwicklung im Bildungswesen. https://doi.org/10.18452/3127

Riemann, R., & Allgöwer, A. (1993). Eine deutschsprachige Fassung des "Interpersonal Competence Questionnaire" (ICQ) [A German version of the "Interpersonal Competence Questionnaire" (ICQ)]. *Zeitschrift für Differentielle und Diagnostische Psychologie*, *14*(3), 153–163.

Schaarschmidt, U., & Fischer, A. W. (2001). *Bewältigungsmuster im Beruf* [Coping patterns at work]. Vandenhoeck & Ruprecht.

Schaeper, H. (2020). The first year in higher education: The role of individual factors and the learning environment for academic integration. *Higher Education*, *79*(1), 95-110. https://doi.org/10.1007/s10734-019-00398-0

Schaeper, H., & Weiß, T. (2016). The conceptualization, development, and validation of an instrument for measuring the formal learning environment in higher education. In H.-P. Blossfeld, J. von Maurice, M. Bayer, & J. Skopek (Eds.), *Methodological issues of longitudinal surveys: The example of the National Educational Panel Study* (pp. 269–292). Springer VS. https://doi.org/10.1007/978-3-658-11994-2_16.

Schiefele, U., Moschner, B., & Hustegge, R. (2002). Skalenhandbuch SMILE-Projekt [Documentation of scales used in the SMILE project]. Bielefeld, Germany. Universität Bielefeld, Abteilung für Psychologie.

Schwarzer, R., & Schmitz, G. S. (1999). Skala zur Lehrer-Selbstwirksamkeitserwartung (WIRKLEHR) [Teacher self-efficacy scale]. In R. Schwarzer & M. Jerusalem (Eds.), *Skalen zur Erfassung von Lehrer-und Schülermerkmalen. Dokumentation der psychometrischen Verfahren im Rahmen der Wissenschaftlichen Begleitung des Modellversuchs Selbstwirksame Schulen* (pp. 60–61). Freie Universität Berlin. http://userpage.fu-berlin.de/~health/self/skalendoku_selbstwirksame_schulen.pdf

Stiftung Universität Hildesheim. (2016). *Fragebogen für Lehrerinnen und Lehrer an Grund- und Förderschulen des Projekts "Einstellungen zur Inklusion in der Schule" (Projekt E1NS).* Unpublished [Questionnaire for teachers at primary and special education schools of the project "Attitudes towards inclusive education in schools"]. Hildesheim. Universität Hildesheim, Institut für Psychologie/Institut für Grundschuldidaktik und Sachunterricht.

Thiel, F., Ophardt, D., & Piwowar, V. (2012). Abschlussbericht des Projekts “Kompetenzen des Klassenmanagements (KODEK). Entwicklung und Evaluation eines Fortbildungsprogramms für Lehrkräfte zum Klassenmanagement” [Report of the research project “Classroom management competencies. Development and evaluation of a teacher training programme in classroom management”]. Freie Universität Berlin. https://doi.org/10.2314/GBV:771646968

Trautwein, U., Husemann, N., Lüdtke, O., Köller, O., Watermann, R., Maaz, K., Nagy, G., & Baumert, J. (2006). *Transformation des Sekundarschulsystems und akademische Karrieren (TOSCA). Dokumentation der eingesetzten Items und Skalen, Welle 2*. Unpublished manuscript [Transformation of the secondary school system and academic careers (TOSCA). Documentation of items and scales, wave 2]. Berlin, Germany. Max-Planck-Institut für Bildungsforschung.

Trommer, M., Schaeper, H., & Fabian, G. (2021). KWReq—a new instrument for measuring knowledge work requirements of higher education graduates. *Journal for Labour Market Research*, *55*, Article 22. https://doi.org/10.1186/s12651-021-00303-2

Vennemann, M., Eickelmann, B., Labusch, A., & Drossel, K. (2021). *ICILS 2018 #Deutschland: Dokumentation der Erhebungsinstrumente der zweiten Computer and Information Literacy Study* [ICILS 2018 #Germany: Documentation of the survey instruments used in the second Computer and Information Literacy Study]. Waxmann. https://doi.org/10.31244/9783830993278

Watt, H. M., Richardson, P. W., Klusmann, U., Kunter, M., Beyer, B., Trautwein, U., & Baumert, J. (2012). Motivations for choosing teaching as a career: An international comparison using the FIT-Choice scale. *Teaching and Teacher Education*, *28*, 791–805. https://doi.org/10.1016/j.tate.2012.03.003

Wenz, S. E. (2020). *Discrimination in education* [Dissertation]. Otto-Friedrich-Universität Bamberg, Bamberg, Germany. https://doi.org/10.21241/SSOAR.67307

Wenz, S. E., Olczyk, M., & Lorenz, G. (2016). *Measuring teachers' stereotypes in the NEPS*. (NEPS Survey Paper No. 3). Bamberg, Germany. Leibniz Institute for Educational Trajectories, National Educational Panel Study. https://doi.org/10.5157/NEPS:SP03:1.0

Weresch-Deperrois, I., Bodensohn, R., & Jäger, R. S. (2009). *KOSTA – Ein Instrument zur Kompetenz- und Standardorientierung in der Lehrerbildung: Skalenhandbuch* [KOSTA – A competence and standard based instrument in teacher training: Scale manual]. Landau, Germany. Universität Koblenz-Landau. http://www.uni-landau.de/schulprakt-studien/091215_Skalenhandbuch_KOSTA1.pdf

Westermann, R., Heise, E., Spies, K., & Trautwein, U. (1996). Identifikation und Erfassung von Komponenten der Studienzufriedenheit [Identifying and assessing components of student satisfaction]. *Psychologie in Erziehung und Unterricht*, *43*, 1–22.

Wettstein, A., Ramseier, E., Scherzinger, M., & Gasser, L. (2017). Unterrichtsstörungen aus Lehrer- und Schülersicht: Aggressive und nicht aggressive Störungen im Unterricht aus der Sicht der Klassen-, einer Fachlehrperson und der Schülerinnen und Schüler [Classroom disturbances from the perspective of teachers and students – Aggressive and nonaggressive disturbances from the point of view of the class teacher, the subject teacher, and the students]. *Zeitschrift für Entwicklungspsychologie und Pädagogische Psychologie*, *48*(4), 171–183. https://doi.org/10.1026/0049-8637/a000159

Wohlkinger, F., Blumenfelder, A. R., Bayer, M., von Maurice, J., Ditton, H., & Blossfeld, H.‑P. (2019). Measuring motivational concepts and personality aspects in the National

Educational Panel Study. In H.-P. Blossfeld & H.-G. Roßbach (Eds.), *Education as a lifelong process: The German National Educational Panel Study (NEPS)* (2nd ed., pp. 155–169). Springer VS. https://doi.org/10.1007/978-3-658-23162-0_9

Year	Publication
2023	Franz, S., Gäckle, S., & Menge, C. (2023). Übergänge von Lehramtsabsolventinnen und -absolventen. Wer verbleibt im ersten Jahr nach Studienabschluss auf dem Weg zur Lehrkraft? [Transitions of teacher education graduates. Who remains on the path to becoming a teacher in the first year after graduation?]. In J. Ordemann, S, Buchholz, & F. Peter (Eds.), *Vielfalt von Bildungsverläufen. DZHW-Jahresschwerpunktthema 2021. HERSS – Higher Education Research and Science Studies*. VS Verlag für Sozialwissenschaften. https://doi.org/10.1007/978-3-658-39657-2_8
2022	Costa, J. (2022). Freiwilliges Engagement als kultureller Erfahrungsraum. Theoretische Perspektiven und deskriptive Befunde zum kulturellen Engagement zukünftiger Lehrkräfte [Voluntary engagement as a cultural space of experience. Theoretical perspectives and descriptive findings on the cultural engagement of prospective teachers]. In A. Scheunpflug, C. Wulf, & I. Züchner (Eds.), *Kulturelle Bildung* (pp. 1–29). Springer VS. https://doi.org/10.1007/978-3-658-35454-1_1
	Costa, J. (2022). Kulturelle Aktivitätsmuster angehender Lehrkräfte. Explorative Befunde zu Aktivitäten von Lehramtsstudierenden außerhalb formalisierter (Lern-)Kontexte [Cultural activity patterns of prospective teachers: Explorative findings on activities of teacher education students outside formal (leaning) contexts]. *Zeitschrift für Pädagogik*, *68*(2), 227–248. https://doi.org/10.3262/ZP2202227
	Costa, J. (2022). *Kultur in der Lehrerinnen- und Lehrerbildungsforschung: Perspektiven auf die Erforschung des kulturellen (Erfahrungs-) Horizonts zukünftiger Lehrkräfte* [Culture in teacher education research: Perspectives on exploring the cultural experiences of future teachers] [Dissertation]. Otto-Friedrich-Universität, Bamberg, Germany. https://doi.org/10.20378/irb-49955
	Franz, S., Fackler, S., & Paetsch, J. (2022). Profiles of pre-service teachers' personality traits and cognitive abilities: Relations with graduation and teacher self-efficacy. *European Journal of Teacher Education.* https://doi.org/10.1080/02619768.2022.2114896
	Gülen, Ş. (2022). Ursachen der Unterrepräsentanz von Lehrkräften mit Migrations-hintergrund an Schulen in Deutschland. Eine theoretische und empirische Analyse des Studienwahlverhaltens [Causes of the underrepresentation of teachers with a migrant background at schools in Germany. A theoretical and empirical analysis of study choice behaviour]. In M. Stock, N. Hodaie, S. Immerfall, & M. Menz (Eds.), *Migration – Gesellschaft – Schule. Arbeitstitel: Migrationsgesellschaft: Pädagogik – Profession – Praktik* (pp. 105–125). Springer VS. https://doi.org/10.1007/978-3-658-34087-2_6
	Hartmann, F. G., Mouton, D., & Ertl, B. (2022). The big six interests of STEM and non-STEM students inside and outside of teacher education. *Teaching and Teacher Education*, 112(3), Article 103622. https://doi.org/10.1016/j.tate.2021.103622
2021	Carstensen, B., & Klusmann, U. (2021). Assertiveness and adaptation: Prospective teachers' social competence development and its significance for occupational well-being. *British Journal of Educational Psychology*, *91(*1), 500–526. https://doi.org/10.1111/bjep.12377
	Gülen, Ş. (2021). *Lehramtsstudium mit Migrationshintergrund: Einflussfaktoren auf die Studienfachentscheidung und den Studienverlauf* [Teacher training with a migrant background: Factors influencing the choice and course of studies]. Springer VS. https://doi.org/10.1007/978-3-658-32882-5

Table A12 Publications based on data from the LAP project

Year	Publication
2021	Hartmann, F. G., & Ertl, B. (2021). Big Five personality trait differences between students from different majors aspiring to the teaching profession. *Current Psychology*. https://doi.org/10.1007/s12144-021-02528-3
	Menge, C., Euler, T., & Schaeper, H. (2021). Überzeugungen und Selbstwirksam-keitserwartungen zum inklusiven Unterricht bei (angehenden) Lehrkräften: der Einfluss von Lerngelegenheiten [Beliefs and perceived self-efficacy of (prospective) teachers regarding inclusive education: The effect of learning opportunities]. *Zeitschrift für Erziehungswissenschaft, 24*(6), 1283-1308. https://doi.org/10.1007/s11618-021-01038-z
	Osada, J.-C., & Schaeper, H. (2021). Individual characteristics of teacher education students: Re-examining the negative selection hypothesis. *Journal for Educational Research Online, 13*(2), 109-131. https://doi.org/10.31244/jero.2021.02.06
	Senkbeil, M., Ihme, J. M., & Schöber, C. (2021). Schulische Medienkompetenz-förderung in einer digitalen Welt: Über welche digitalen Kompetenzen verfügen angehende Lehrkräfte? [Dissemination of media literacy at school in a digital world: Are teacher candidates digitally competent?]. *Psychologie in Erziehung und Unterricht*, *68*(1), 4–22. https://doi.org/10.2378/peu2020.art12d
2020	Costa, J., & Drechsel, B. (2020). Künstlerisch-ästhetische Praxen angehender Lehrkräfte: Befunde einer quantitativen Sekundärdatenanalyse [Artistic-aesthetic practices of prospective teachers: Findings from a quantitative secondary data analysis]. In S. Timm, J. Costa, C. Kühn, & A. Scheunpflug (Eds.), *Kulturelle Bildung: Theoretische Perspektiven, methodologische Herausforderungen, empirische Befunde* (pp. 129–146). Waxmann. https://doi.org/10.31244/9783830991502
	Neugebauer, M. (2020). Leistungsmerkmale [Performance characteristics]. In C. Cramer, J. König, M. Rothland, & S. Blömeke (Eds.), *Handbuch Lehrerinnen- und Lehrerbildung* (pp. 798–803). Julius Klinkhardt. https://doi.org/10.35468/hblb2020-098
2019	Menge, C., & Schaeper, H. (2019). Berufliche Selbstregulation von Lehrkräften: Überprüfung eines Kurzinstruments [Teachers' occupational self-regulation: Validation of a short measurement instrument]. *Zeitschrift für Erziehungswissen-schaft, 22*(6), 1489-1513. https://www.doi.org/10.1007/s11618-018-0851-x
	Pozas Guajardo, M., & Schneider, C. (2019). Shedding light on the convoluted ter-rain of differentiated instruction (DI): Proposal of a DI taxonomy for the heterogeneous classroom. *Open Education Studies*, *1*(1), 73–90. https://doi.org/10.1515/edu-2019-0005
	Rochnia, M., Trempler, K., & Schellenbach-Zell, J. (2019). Vergleich der Forschungs- sowie Praxisorientierung zwischen Lehramts- und Medizinstudium [Comparison of research and practice orientation between teacher and medical training]. *Zeitschrift für empirische Hochschulforschung*, *2*(2), 123–138. https://doi.org/10.3224/zehf.v3i2.03
2017	Besa, K.-S., & Vietgen, S. (2017). Repräsentanz, Integration und Abbruchintentio-nen von Studierenden mit Migrationshintergrund in Lehramtsstudiengängen: Eine Analyse anhand der Daten des Nationalen Bildungspanels (NEPS) [Representative-ness, integration and dropout intentions of immigrant students in teacher pre-paration programmes: An analysis of NEPS data]. *Beiträge zur Lehrerinnen- und*

	Lehrerbildung, 35(1), 195-206. https://doi.org/10.25656/01:16806

Table A12 cont.
