# Supplementary material for: The German Panel of Teacher Education Students: Surveying (Prospective) Teachers from Higher Education into Working Life
Source: J Open Psychol Data. 2023 May 10;11:7. doi: 10.5334/jopd.76 (PMC12270278; doi:10.5334/jopd.76)
Supplement: PR File 1. — Peer Review History. [file jopd-11-76-pr1.zip › pr1-jopd-76_wolter/Reviewer-comments_answers_R1.pdf]

The German Panel of Teacher Education Students: Surveying (prospective) teachers from higher education into working life

Answers to the comments

Comment	Answer
<u>General comments on the revision</u><br><br>We would like to thank the editors for the opportunity to revise and resubmit the manuscript. We are very grateful for the critical and constructive comments made by both editor and reviewers. We carefully considered the annotations and often responded to them by modifying the paper. However, sometimes we had reasons not to comply with a comment. We are convinced that incorporating the editor's and reviewers' feedback improved the paper. And we hope that our reasons for not following some suggestions are convincing.<br><br>Most changes are tracked so that they can be easily recognised. Major reorganisations of the paper such as moving tables into the appendix are not tracked but described in the answers to the comments. As the tables from Chapter 2.5.2 were moved to the Appendix, the references for the sources of the instruments were also moved to the Appendix and deleted from the main reference list.<br><br>In addition to the adjustments already mentioned and documented below, other minor changes were necessary. These are due to the recently published new SUF version (wave 17) and to standardisations in citation and terminology. These changes are tracked as well.	
**Editor**	
<u>#1 (NEPS – SC5 – LAP)</u><br>The distinction between NEPS, NEPS SC5 and this LAP study could be made even clearer for an international audience in section 1 and 2.1. One idea could be to start hierarchically from the most general level—that is, very briefly describe NEPS, then SC5 and then make clear that LAP is a kind of "add-on" project, say when it started and ended (compared to when NEPS as a whole started and when SC5 started). This information is included in the manuscript, but I think the distinction of these levels of the NEPS should be presented in one place and in consistent way.	We have attempted to describe the relationship between NEPS, SC5 and LAP more precisely. On the one hand, we have added a sentence at the end of the second paragraph of Chapter 1: "In terms of design, the LAP study is fully integrated into NEPS SC5; in terms of constructs and survey instruments, it has complemented NEPS SC5 since 2014 (wave 8)."<br><br>On the other hand, more detailed information has been newly introduced in Chapter 2.1 at the end of the fourth paragraph: "All survey instruments were basically also addressed to (prospective) teachers. They already open up a wide range of possibilities for research on teacher training and the teaching profession. When the LAP project started, an additional survey programme specifically for (prospective) teachers has been implemented in the NEPS SC5 surveys since wave 8 in autumn 2014, with the LAP study fully integrated into the survey design."
<u>#2 (data for which field of psychology?)</u><br>Since this journal focuses on data for the domain of psychology, the authors should emphasize the reuse potential of the data for the fields of educational, developmental and applied/organizational psychology a bit more clearly. "Psychological factors" are mentioned in several parts of the manuscript, but it could be made clearer which subfields of psychology and psychological research questions researchers could tap into with this dataset. "	At the end of Chapter 1, we clarified that the LAP data can be mainly used for answering research questions from educational psychology: "These examples show that the LAP data can be used to address a wide range of research questions, particularly in educational psychology."

Comment	Answer
<u>#3 (suggestion for shortening the paper)</u><br>I agree with the reviewers that the manuscript is quite long, but I think this is warranted given the complexity of the study. If the authors wanted to shorten the manuscript, however, I think the Tables in 2.5.2 could be moved to the Appendix and the description of instruments in the text (which is very detailed) could focus more strongly on psychological factors and point to the appendix for the full overview.	We have followed the suggestion and moved the tables in Chapter 2.5.2 to the Appendix. As a consequence, the references for the sources of our instruments were also moved to the Appendix and deleted from the main reference list.
<u>#4 (suggestion for shortening the paper)</u><br>The publications mentioned in 2.8 could also be put in an Appendix table.	We have also followed this recommendation and added newly published papers to the list of publications.
**Reviewer A**	
<u>#1 (potential for shortening the paper)</u><br>“[..] there is potential for shortening, e.g. Chapter 2.1 Detailed description of NEPS, Chapter 2.6 Quality control.”	Since important information on the study design is given in Chapter 2.1, we do not see much potential for shortening here. However, in Chapter 2.6 we deleted or shortened several paragraphs.
<u>#2 (relationship NEPS – SC5 – LAP)</u><br>The description of the data changes the perspective from LAP as part of NEPS to NEPS with additional survey LAP. Particularly in the chapter “study design”, in NEPS is first discussed in general terms. As a result, it is not always clear to which study the waves mentioned refer (NEPS general, NEPS SC5 or LAP). The assignment to the respective study could be supplemented. It would also be helpful to mention in the introduction that LAP (or SC5?) covers the period from 2010 to 2020 with 19 survey waves. It remains unclear why Table 2 only includes waves 8-19 of the 19 waves.	As noted above (answer to comment #1 of the editor), we provided some additional explanation on how the NEPS, NEPS SC5 and LAP are related. We hope that it is now clearer that certain waves cannot be assigned to SC5 or LAP, but that the two studies differ mainly in the survey programme. The LAP target persons receive the same questionnaires and tests as the SC5 sample, but in addition they are asked teacher- or teaching-specific questions from wave 8 onwards.<br><br>Information on the number of waves and the observation period is now prominently placed in Chapter 1 at the end of paragraph 2.<br><br>In Table 2 we only considered waves from 8 to 17 since these waves contain filtering variables generated to lead through the teacher-related questions. As it has been mentioned previously in the paper, LAP-specific questions have been introduced from wave 8 onwards. This means that before wave 8 it was not necessary to ask filtering questions. We modified a sentence at the first mention of Table 2 to make this clearer and to prevent possible misunderstandings.
<u>#3 (concept of competence)</u><br>“The concept of competence used in the LAP study follows Baumert and Kunter’s (2013) multidimensional competence model, which includes both cognitive (e.g., professional knowledge) and non-cognitive (e.g., beliefs, motivation) dimensions. Since it was not possible to measure professional knowledge, the study focuses on beliefs (e.g., beliefs about teaching and learning), motivational orientations (e.g.,	We tried to reduce the confusion by making clearer that we used the model of Baumert and Kunter to conceptualise teachers’ professional competence, that we couldn’t measure professional knowledge of (prospective) teachers, but that as part of the general SC5 testing programme other cognitive competencies were assessed. We hope that adding the sentence “However, as part of the general NEPS SC5 testing programme, a selection of basic cognitive competencies, such as scientific literacy (see Chapter 2.5.2) was assessed.” serves our purpose.

Comment	Answer
motivation for choosing teacher education, teaching-related self-efficacy), and occupational self-regulation. In addition, data on teaching-related abilities in information and communication technologies (ICT) was collected using self-report instruments."<br><br>=> This text passage was not clear to me and also led to confusion in the following passages in which the competences surveyed were dealt with. According to Baumert and Kunter's model, professional knowledge is the cognitive dimension, an example of a sub-dimension would be "pedagogical content knowledge". However, other cognitive competences are surveyed in the study that are not part of Baumert and Kunter's model (and also go beyond ICT skills). Perhaps the information, that competences in the areas of XXX are collected beyond Baumert and Kunter's model, can be added to the above paragraph.	
#4 (inconsistent use of terms)<br>NEPS Starting Cohort First-Year Students vs SC5 vs NEPS SC5 o initial sample vs. initial group vs. LAP basic sample.	Throughout the paper we now refer to NEPS Starting Cohort "First-Year Students" as NEPS SC5. A corresponding footnote is included at the first mention of NEPS SC5 in Chapter 1. We have also made sure that the other terms are used consistently.
#5 (research questions/research potential)<br>The description of potential research questions is very valuable. In the text, it could be made more explicit which content areas can be analysed with the data presented, what has already been analysed and what further analysis potential is. In a few places, the amount of examples gave the impression that certain areas were interesting but had already been extensively researched. Eventually, the information on implemented research projects and potentials in the Background chapter could be shortened and corresponding information could be bundled in the chapters "Potentials" and "Existing use of data".	Thank you for this feedback and the suggestion. In the section on research potentials we highlighted some of the many topics that the rich data set of LAP in combination with NEPS SC5 allows. However, to thoroughly follow the reviewer's suggestion an extensive literature review of existing analyses with LAP data is needed and is from our point of view beyond the scope of our manuscript.<br><br>While there are already studies on topics such as specific characteristics of teachers who enter the teaching profession, aspects of preparatory service, and teachers' exhaustion in the teaching profession, other topics with regard to cooperation, further education or the impact of school leadership, for example, are scarcely researched with LAP data until now. We tried to elaborate on the variety of information and consequently the research potentials throughout the paper and thereby hope to have opened the perspective for this data set for various research questions.
#6 (comprehensibility for foreign researchers)<br>The text takes into account the international focus of the journal in many places and describes particularities of the German school and university system. Nevertheless, the comprehensibility for researchers that are not common to the German system could be critically reviewed in some places. E.g.	We explained the meaning of the term "non-traditional students" by adding "i.e., students without a school-leaving certificate qualifying for higher education" (Chapter 2.4, paragraph 1).<br><br>Although in Germany there is a substantial overlap between school types and tracks, these terms are not identical. We therefore have changed the corresponding sentence and chosen a more precise wording: In the initial sample, 13 % of the teacher education students had taken

Comment	Answer
o “However, first- year students studying at higher education institutions run by federal ministries or federal states for members of their own public services were excluded. During sampling, special emphasis was placed on students with non-traditional entrance qualifications. „<br>o “Since in Germany the school system is divided into different tracks, the various school types are also mirrored in different teacher education programmes. In the initial sample, 13 % of the teacher education students had taken up a training course for primary education, 19.5 % for lower secondary education (Haupt- und Realschule), 54.5 % for upper secondary education (Gymnasium), 5 % for special education (Sonder-/ Förderschule), and 6.5 % for vocational education (Berufsschule). One and a half per cent of the teacher education students stated that their degree programme did not differentiate between school types. As is common in teacher education programmes, most students are female (75.5 %).” =>Herausforderungen “school type” vs. “track”. Hier wird “school type” und “track” vermischt, gleichzeit sind “school types” wie “Mittelschule und Gesamtschule” nicht aufgeführt.	up a training course for primary education, 19.5 % for lower secondary education (excluding lower secondary education at a *Gymnasium*), 54.5 % for upper secondary education (including lower secondary education at a *Gymnasium* but excluding vocational education), 5 % for special education, and 6.5 % for vocational education.
#7 (links)<br>Additional materials and information are linked and are very helpful. Concrete links where and how data can be downloaded or requested would be a valuable addition.	The requested links to where and how our data can be downloaded were added in Chapter 3.1.
**Reviewer B**	
#1 (abstract)<br>The abstract does not make clear what the goals of this study are. It is indicated that the longitudinal study represents the perspective of prospective teachers, following their professional and competence development from teacher education to the first years of professional life. It is not made clear that the manuscript describes the survey instruments and their potential for use. It is indicated that it is a longitudinal study, but a number of survey waves is not mentioned.	We modified the first sentence of the abstract: “This paper describes the design, survey instruments, data, and their potential for use of a longitudinal study of (prospective) teachers in Germany that follows their professional and competence development from teacher education into the first years in the teaching profession.” In addition, we included the information on the number of waves and the observation period at the end of the abstract. Due to these additions, the abstract now exceeds 100 words.
#2 (background)<br>In the introduction, the background of the study is described. In addition, the overview of the structure is presented and then the theo-	Indeed, there is some overlap between the chapters. However, in Chapter 1 we elaborate on the research questions and theoretical foundations of the LAP study and embed the constructs to be measured in the theoretical considerations, thus providing a theoretical rationale for the selection of certain constructs. In Chapter 2.5.2 we concentrate on measurement issues and

Comment	Answer
retical background for the use of the survey instruments. The description of the survey instruments is partly redundant with chapter 2.5.2.	refer to theory primarily in order to explain our measurement approach. We think that this division of labour works quite well and hope that the few repetitions are acceptable.
#3 (Cronbach's alpha)<br>In chapter 2.5.2 it is recommended to add additional information on Cronbach's alpha to the tables in order to be able to consider the internal consistency of the scales.	We are very sorry that right now we cannot provide information on Cronbach's alpha. We are preparing an extensive documentation of all questions and scales measured in the LAP study, which will include Cronbach's alpha, item test correlation, and other statistics. We have already pointed this out in the text (Chapter 2.5.2, paragraph 1, last sentence).
#4 (list of publications in Chapter 2.8)<br>The listing of publications on prospective teachers in the middle of text is quite confusing. It is advisable to define the topic blocks and to cite the specified publications accordingly. Alternatively, this could be integrated into chapter four, which deals with the potential use of the data.	The list of publications based on LAP data has been moved to the Appendix. Topic-related references to publications using LAP data are given in Chapter 1.
#5 (missing, implausible, inconsistent values)<br>There is no information in the manuscript on how missing values were dealt with in the study. It is recommended to include an additional chapter on this, with information on whether there are missing values, how they are defined in the study, if applicable, which categories of missing values there are and what their significance is. Include, if applicable, how values outside the intended range of values (wild codes) or implausible (outliers) or inconsistent values in individual variables are handled.	We have included a paragraph on missing values in Chapter 3.10. However, we did not give a detailed description of the numerous types of missing values that occur in the data, as this would go beyond the scope of our paper. Instead, we refer to other publications on this topic that provide further information on how missing values were computed and how to deal with them.
#6 (weights)<br>The manuscript does not include information on whether sampling weights were created. The information may need to be added in the extra chapter to include a description of how the weights were created and how they can be used.	We have included a paragraph on weights in Chapter 3.10. Again, we did not give a detailed description of the estimation and usage of the weights but referred to other papers dealing with this topic.
#7 (summary)<br>Although the data presented is very interesting, I think the authors have not yet succeeded in making it clear, especially at the beginning of the manuscript, what the aims of the paper are. The manuscript provides an overview of the survey instruments used in the study and how they can be used. This intention is not clearly shown at the beginning of the manuscript.	As already mentioned, we changed the abstract to make the purpose of the paper clearer. In addition, we also modified the beginning of Chapter 1.
