# Supplementary material for: The German Panel of Teacher Education Students: Surveying (Prospective) Teachers from Higher Education into Working Life
Source: J Open Psychol Data. 2023 May 10;11:7. doi: 10.5334/jopd.76 (PMC12270278; doi:10.5334/jopd.76)
Supplement: PR File 1. — Peer Review History. [file jopd-11-76-pr1.zip › pr1-jopd-76_wolter/Reviewer-comments_answers_R2.pdf]

The German Panel of Teacher Education Students: Surveying (prospective) teachers from higher education into working life
Revision 2

Thank you for submitting the revised version of your manuscript " The German Panel of Teacher Education Students: Surveying (prospective) teachers from higher education into working life" to our JOPD special collection "Data for Psychological Research in the Educational field". I think the revision addressed the reviewers' points very convincingly. After having another look at the manuscript and after a discussion within the editorial team (with the goal of consistency across the manuscripts within the special issue), I have two more points that should be addressed before publication:

(1) The background section already includes several statements tapping into the reuse potential of the data—that is, possible research questions that could be addressed with the data (e.g. "Research topics that can be addressed entail the question of how the perceived quality of the preparatory service affects emotional exhaustion, job satisfaction, teaching practices, and teachers' self-efficacy."). We would encourage you to check whether such statements can be moved to Section 4 (reuse potential) thereby possibly also shortening the background section and removing redundancies.

(2) We decided to require a more comprehensive list of data objects within the data package for sections 3.2 to 3.4 to be included in the manuscript rather than just as a reference. An option would be to include a table that includes the filenames and formats of all relevant datasets and also documentation files, but other options (e.g. Bullet Points) are also possible. You can also look at the already published articles of the special issues for examples (e.g., von Maurice & Will on the ReGES data or Martin et al on PIAAC-L; https://openpsychologydata.metajnl.com/articles/10.5334/jopd.74/).

Thank you very much for your valuable comments and for giving us the opportunity to present the LAP data to the scientific community.
We made the modifications you suggested. Before altering the manuscript, we accepted all changes made during the first revision and tracked the new modifications (except the new tables).

Research questions not mentioned in chapter 4 ("reuse potential") were moved from chapter 1 to chapter 4. Research questions already addressed in chapter 4 were deleted in chapter 1. As a result of these changes, chapter 1 could be shortened by about 17 per cent. In chapter 4, we deleted passages/sentences that we considered less important or redundant to make room for the additional information on the research questions. The modifications in chapter 1 also led to the deletion of three references.

As suggested, we included a new table with an overview of the SUF datasets (Table 3) and of the relevant documents and materials (Table 4).

We also added a sentence to the acknowledgements section to thank the reviewers and editors for their comments and suggestions.
In addition to the changes mentioned above, we made some minor corrections to inconsistencies and mistakes we have only just noted (in the main text and also in the appendix).
